# Reactive Oxygen Species and Endothelial Ca^2+^ Signaling: Brothers in Arms or Partners in Crime?

**DOI:** 10.3390/ijms22189821

**Published:** 2021-09-10

**Authors:** Sharon Negri, Pawan Faris, Francesco Moccia

**Affiliations:** Laboratory of General Physiology, Department of Biology and Biotechnology “L. Spallanzani”, University of Pavia, 27100 Pavia, Italy; sharon.negri01@universitadipavia.it (S.N.); faris.pawan@unipv.it (P.F.)

**Keywords:** endothelial cells, Ca^2+^ signaling, reactive oxygen species, hydrogen peroxide, glutathione, superoxide anion, InsP_3_ receptors, transient receptor potential channel, STIM, Orai

## Abstract

An increase in intracellular Ca^2+^ concentration ([Ca^2+^]_i_) controls virtually all endothelial cell functions and is, therefore, crucial to maintain cardiovascular homeostasis. An aberrant elevation in endothelial can indeed lead to severe cardiovascular disorders. Likewise, moderate amounts of reactive oxygen species (ROS) induce intracellular Ca^2+^ signals to regulate vascular functions, while excessive ROS production may exploit dysregulated Ca^2+^ dynamics to induce endothelial injury. Herein, we survey how ROS induce endothelial Ca^2+^ signals to regulate vascular functions and, vice versa, how aberrant ROS generation may exploit the Ca^2+^ handling machinery to promote endothelial dysfunction. ROS elicit endothelial Ca^2+^ signals by regulating inositol-1,4,5-trisphosphate receptors, sarco-endoplasmic reticulum Ca^2+^-ATPase 2B, two-pore channels, store-operated Ca^2+^ entry (SOCE), and multiple isoforms of transient receptor potential (TRP) channels. ROS-induced endothelial Ca^2+^ signals regulate endothelial permeability, angiogenesis, and generation of vasorelaxing mediators and can be exploited to induce therapeutic angiogenesis, rescue neurovascular coupling, and induce cancer regression. However, an increase in endothelial [Ca^2+^]_i_ induced by aberrant ROS formation may result in endothelial dysfunction, inflammatory diseases, metabolic disorders, and pulmonary artery hypertension. This information could pave the way to design alternative treatments to interfere with the life-threatening interconnection between endothelial ROS and Ca^2+^ signaling under multiple pathological conditions.

## 1. Introduction

The vascular endothelium lines the innermost layer of the entire circulatory system and serves as a signal transduction platform that senses and integrates mechanical forces (e.g., pulsatile stretch and shear stress), chemical cues (e.g., hormones, growth factors, and autacoids), and thermal stimuli (e.g., increases in body temperature) to finely tune virtually all cardiovascular functions [1,2,3]. Therefore, peripheral vasculature is endowed with multiple progenitor cell niches that release on demand, e.g., upon an ischemic insult or a traumatic injury, endothelial colony forming cells (ECFCs) to replace damaged endothelial cells [4]. An increase in intracellular Ca^2+^ concentration ([Ca^2+^]_i_) is the most versatile signaling pathway whereby either a subtle or gross change in extracellular microenvironment may instruct endothelial cells and circulating ECFCs to perform a specific task to maintain cardiovascular homeostasis [1,2,5,6,7,8,9]. Distinct spatiotemporal endothelial Ca^2+^ signals tightly regulate different functions such as nitric oxide (NO) release [10,11,12] and endothelium-dependent hyperpolarization (EDH) [13], vascular permeability [14,15] and repair [16,17], platelet aggregation and blood coagulation [18,19], leukocyte/lymphocyte infiltration [20,21,22,23], neurovascular coupling [24,25], wound healing [16,17], angiogenesis [5,26], and vasculogenesis [27]. An aberrant, i.e., resulting either from intracellular Ca^2+^ overload or by the dismantling of a specific oscillatory Ca^2+^ pattern, or insufficient elevation in [Ca^2+^]_i_ may lead to endothelial dysfunction and therefore severely compromise cardiovascular homeostasis, as reported in atherosclerosis [28], hypertension [29,30], pulmonary artery hypertension (PAH) [31], type 2 diabetes [8,32,33], aging [34], inflammatory disorders [21,22,35,36,37], Alzheimer’s disease, and cerebrovascular dysfunction [34,38,39,40,41]. Therefore, the endothelial [Ca^2+^]_i_ must be tightly regulated by a sophisticated network of membrane receptors, ion channels, pumps, transporters, and cytosolic Ca^2+^ buffers to prevent the onset of inappropriate Ca^2+^ signals that could hamper the cardiovascular system [1,2,8,42,43].

**Figure 1:**
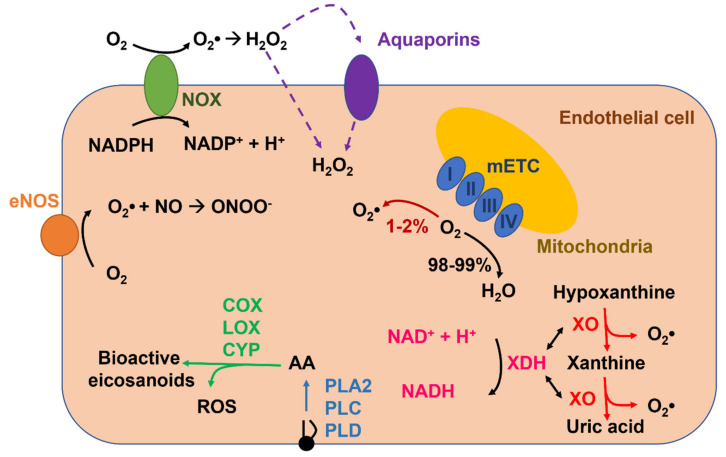
Major mechanisms of ROS production in vascular endothelial cells. The enzyme NADPH oxidase (NOX; green) catalyzes the transfer of an electron from NADPH to O_2_, generating O_2_•^−^ in the extracellular space. O_2_•^−^ is rapidly dismutated into H_2_O_2_, which may freely diffuse across the plasma membrane or enter the cytosol through aquaporins (purple). O_2_•^−^ is continuously generated in the mitochondria (right) by members (blue) of the electron transport chain machinery (mETC; blue) in the inner mitochondrial membrane. 1%-2% of the O_2_ consumed is estimated to be converted into O_2_•^−^ and not into H_2_O_2_. A fraction of this O_2_•^−^ can then leak to the cytoplasm through the VDACs in the outer mitochondrial membrane. During the oxidation of hypoxanthine to xanthine and xanthine to uric acid, XDH catalyzes the reduction of NAD+ to NADH, whereas XO catalyzes the reduction of O_2_ to O_2_•^−^ and not into H_2_O_2_. Arachidonic acid, which may be produced upon cleavage of glycerophospholipids on the plasma membrane by PLD, PLC, and PLA2, may generate ROS as secondary byproducts during its conversion into an array of bioactive eicosanoids by COXs, LOXs, and CYPs. Finally, eNOS (orange) releases NO in the presence of BH_4_ (coupled eNOS), while it produces O_2_•^−^ in the absence of BH_4_ (uncoupled eNOS).

Reactive oxygen species (ROS), which are produced in vascular endothelial cells during their metabolic activity or upon extracellular stimulation (Figure 1), might also serve as a double-edged sword [44,45,46]. Endothelial ROS signaling is exploited by mechanical and chemical cues to regulate a number of vascular functions that often overlap with those controlled by Ca^2+^, e.g., EDH [47], vascular permeability [48], leukocyte infiltration [49], platelet aggregation [50], gene expression [51], angiogenesis [45,52], and vasculogenesis [53]. Like Ca^2+^, deregulated ROS signaling impairs endothelial-mediated functions, thereby engendering potentially catastrophic consequences for the cardiovascular system [22,31,36,38,54,55,56,57,58,59]. The existence of a functional crosstalk between endothelial Ca^2+^ and ROS signaling is further strengthened by the evidence that ROS may stimulate an increase in [Ca^2+^]_i_ [6,60,61,62] and that, vice versa, intracellular Ca^2+^ signals may boost endogenous ROS production in vascular endothelium [57,63,64]. Herein, we highlight the main mechanisms whereby intracellular ROS elicit endothelial Ca^2+^ signals by regulating inositol-1,4,5-trisphosphate (InsP_3_) receptors (InsP_3_Rs), sarco-endoplasmic reticulum Ca^2+^-ATPase 2B (SERCA2B), two-pore channels (TPCs), store-operated Ca^2+^ entry (SOCE), and several isoforms of transient receptor potential (TRP) channels. In parallel, we illustrate the multiple vascular functions regulated by ROS-induced endothelial Ca^2+^ signals. Finally, we describe how ROS-dependent endothelial Ca^2+^ signals could be exploited for therapeutic purposes and, vice versa, how excessive ROS production can result in cardiovascular disorders through an aberrant elevation in endothelial [Ca^2+^]_i_.

## 2. ROS Production and Elimination in Endothelial Cells

ROS is a term used to describe several reactive molecules deriving from the incomplete reduction of oxygen, such as superoxide anion (O_2_•^−^), hydroxyl anion (OH•), and hydrogen peroxide (H_2_O_2_) (Figure 1). They are continuously produced and transformed in response to several endogenous and exogenous stimuli under physiopathological conditions. ROS are involved in several biological processes such as cellular growth, immune response, embryogenesis, spermatozoa capacitation, and transcription factor activation [44,65]. Furthermore, ROS regulate vascular functions (e.g., vasodilatation, vasoconstriction, angiogenesis, migration, and apoptosis) [41,44,45,46]. Thus, there is a finely regulated balance between ROS production and ROS degradation [46]. Indeed, when ROS production exceeds the cellular antioxidant defenses (i.e., the so-called toxic threshold), the cells undergo oxidative stress, which may cause DNA damage, protein and lipid modifications, energetic deficit, and cell death [46,65]. Conversely, a temporal and spatial regulated production of ROS, in response to physiological and pathological surges, reversibly mediate the activation or inhibition of molecular targets (e.g., ion channels, transmembrane proteins, and transcriptional factors), by triggering the so-called redox signaling [66]. In this view, different ROS species are characterized by different reactivity and different specificity for their target. The most reactive ROS is OH•, which has a short lifetime. Indeed, O_2_•^−^ is rapidly transformed in H_2_O_2_ either spontaneously or by superoxide dismutase (SOD), and it is featured by a low selectivity toward molecular targets [44]. On the other hand, H_2_O_2_ presents all the characteristics to be a good second messenger by inducing the redox signaling. In accord, it is featured by a longer half-time life; for this reason it may activate targets that are far from the production site [67].

It has long been known that a moderate amount of endothelial ROS recruit specific signaling pathways, including those controlling angiogenesis, permeability, and vasorelaxation, while aberrant ROS production results in endothelial dysfunction [44,45,46,68]. ROS mainly operate by modifying the cysteine thiols in the regulatory domain or in the active site of their molecular target through the S-glutathionylation of protein thiolate anions, or by oxidating the iron–sulfur cluster-containing centers [46]. There are several sources of ROS in the endothelium (Figure 1), which include enzymatic systems, such as NADPH oxidases (NOXs), xanthine oxidoreductase, uncoupled endothelial NO synthase (eNOS), and the mitochondrial respiratory chain. Furthermore, endothelial ROS production may also arise downstream of arachidonic acid metabolism via lipoxygenase (LOX), cyclooxygenases (COX) or cytochrome P450 (CYP) (Figure 1) [69]. ROS production by these sources requires the reduction of molecular oxygen (O_2_) to O_2_•^−^ through a one-electron transfer process. O_2_•^−^ is highly unstable and is rapidly dismutated into H_2_O_2_ as described above [69,70]. Here, we summarize the main mechanisms responsible for ROS production in endothelial cells.

### 2.1. NADPH Oxidase-Mediated ROS Production in Endothelial Cells

Growing evidence indicates that NOX plays a major role in ROS production in vascular cells, including endothelial cells [71,72,73] and ECFCs [53]. NOXs represent a large family of 7 transmembrane enzymes (NOX1-5 and DUOX1-2). All the isoforms are characterized by 6 transmembrane alpha helices and cytosolic NH_2_- and COOH-extremities. Moreover, NOXs are the only enzymes that generate ROS as a primary product in a tightly regulated manner; indeed, they comprehend a catalytic core and several regulatory subunits (i.e., p22^phox^, p47^phox^, p67^phox^, p40^phox^, and Rac1). NOXs mediate the transfer of electrons from NADPH to O_2_ across biological membranes in order to generate primarily O_2_•^−^, which can subsequently be dismutated into H_2_O_2_ (Figure 1) [46,69]. These proteins may be expressed either on the plasma membrane or on endogenous organelles, such as mitochondria, endosomes, the nucleus, and the ER, and their localization is fundamental to dictate whether ROS formation occurs in the extracellular milieu or in the cytoplasm [46]. NOX4 is the most abundant isoform expressed in endothelial cells [46,65,69] and ECFCs [53], and it is responsible for maintaining basal vascular ROS production during physiological metabolic activity [73]. NOX4 is the only isoform that is constitutively activated at a low level because it does not need to combine with any accessory subunits and is only regulated by its expression levels [65,69]. For instance, endothelial NOX4 is upregulated in response to ischemia/hypoxia, starvation, and transforming growth factor-β (TGF-β) [65]. Intriguingly, NOX4 mainly releases H_2_O_2_ rather than O_2_•^−^ and, therefore, is more suitable to regulate endothelial redox-sensitive pathways since H_2_O_2_ is more stable, although it is less freely diffusible as once thought [74] and does not interact with NO to dismantle NO-dependent signaling [65]. In contrast, NOX2, which is also quite abundant in vascular endothelium, is recruited downstream of G_q/11_ protein coupled receptors (G_q/11_PCRs) or tyrosine kinase receptors (TKRs) on the plasma membrane and by metabolic mediators, such as glucose and insulin [65], whereas NOX5 is engaged by an increase in [Ca^2+^]_i_ [64]. However, endothelial NOXs-derived ROS could also transduce the physical stimuli induced by blood flow [75]. Finally, NOXs-derived ROS could result in further ROS release from multiple endogenous sources, including mitochondria, xanthine oxidoreductase, and eNOS, and thereby enhance the oxidative stress imposed to vascular endothelial cells [65,76]. Finally, in the presence of iron (Fe^2+^), H_2_O_2_ produced by NOX activity undergoes the Fenton reaction and forms OH•, an inducer of lipid peroxidation [70,77]. Intriguingly, endogenous products of lipid peroxidation, such as 4-hydroxy-2-nonenal (4-HNE), may target some endothelial TRP channels [60,78,79].

### 2.2. Xanthine Oxidoreductase

Xanthine oxidoreductase (XOR) exists in two interconvertible isoforms, i.e., xanthine oxidase (XO) and xanthine dehydrogenase (XDH) [80]. XOR is a molybdenum-containing iron-sulfur flavoprotein of about 300 kDa that catalyzes the reduction of hypoxanthine and xanthine into uric acid during purine catabolism by generating H_2_O_2_ and O_2_•^−^ as secondary byproducts (Figure 1) [81]. More precisely, XDH reduces NAD+ to NADH, while the XO isoform reduces O_2_ to O_2_•^−^ and H_2_O_2_ [82]. Thus, the balance between XO and XDH is fundamental to determine the amount of ROS generated by these isoforms [69]. XDH is the main isoform detected in well-perfused tissues, and it is converted into XO through several processes, including proteolysis and/or thiol oxidation under multiple pathological conditions, such as ischemia, hypoxia, and inflammation [46,82]. For instance, XO is the main source of ROS during the ischemia-reperfusion injury [82]. As discussed elsewhere [46], XDH is released in circulation by damaged epithelial cells, such as those of mammary gland, intestine, and liver, and is then converted into XO, which ultimately binds to vascular endothelial cells glycosaminoglycans. This induces severe endothelial injury during liver and intestine disorders [82]. Finally, XDH conversion to XO may be increased by oxidative stress through NADPH oxidase [83]. Furthermore, XOR may directly donate electrons to O_2_, thereby directly producing H_2_O_2_ [70,77].

### 2.3. Uncoupled eNOS

NO is a gasotransmitter that regulates multiple endothelial-dependent functions, ranging from the regulation of vascular tone to angiogenesis [9,84,85]. Three different isoforms of NOS have been described in mammals: endothelial NOS (eNOS or NOS3), neuronal NOS (nNOS or NOS1), which are constitutively activated, and inducible NOS (iNOS or NOS2) that is activated in response to an inflammatory status or to proangiogenic stimuli. All the isoforms are flavin- and heme- proteins that assemble as homodimers and require multiple cofactors (i.e., tetrahydrobiopterin or BH_4_, L-arginine, and COQ10) to maintain the monomeric structure that is necessary to produce NO. NOSs serve as oxidoreductases that catalyze flavin-dependent electron transfer from the COOH-terminal bound NADPH to the heme iron and BH_4_ that are located on the NH_2_ terminus, thereby oxidizing L-arginine to L-citrulline and forming NO (coupled NOS) [46]. This reaction requires two steps. First, NOS hydroxylates L-arginine to *N*^ω^-hydroxy-L-arginine; then, it oxidates *N*^ω^-hydroxy-L-arginine to L-citrulline and NO [86]. The shortage of substrates and/or cofactors, mainly BH_4_, may uncouple eNOS from NO release, thus limiting NO bioavailability, and lead to the reduction of O_2_ to O_2_•^−^ (uncoupled eNOS) [46,69]. The ratio between NO and O_2_•^−^ formation is a crucial determinant of endothelial cell fate, since an excess of O_2_•^−^ rapidly reacts with NO by generating peroxynitrite (ONOO^-^), which further dampens NO signaling and causes endothelial dysfunction [87,88]. Uncoupled eNOS-dependent O_2_•^−^ production has been associated to many cardiovascular diseases that present endothelial dysfunction, such as diabetes, hypertension, and atherosclerosis [89,90,91]. Interestingly, NOX-dependent ROS production reduces BH_4_ bioavailability upon oxidation to BH_2_, thereby favoring eNOS uncoupling and enhancing the oxidative stress imposed on endothelial cells [82].

### 2.4. Mitochondria

Mitochondria represent the main intracellular ROS source, mainly via the mitochondrial electron transport chain machinery (mETC), which is situated in the inner mitochondrial membrane [46]. The mETC is composed of 5 complexes: NADH-quinone oxidoreductase (Complex I), succinate dehydrogenase (Complex II), coenzyme Q-cytochrome C oxidoreductase (Complex III), cytochrome C oxidase (Complex IV), and ATP synthase (Complex V) [52]. The Krebs cycle, which is a Ca^2+^-dependent process [92], generates FADH_2_ or NADH that serve as electron donors for four complexes (I-IV) in the mETC, each catalyzing the reduction of O_2_ to H_2_O through a single-electron transfer reaction [46]. Indeed, 1%-2% of the O_2_ consumed is estimated to be converted into ROS and not into water [69]. In this view, mitochondrial ROS are not only a byproduct of oxidative metabolism, but they may have a signaling function within the mitochondria or between other organelles [46,93]. Moreover, ROS may be produced in the intermembrane space by the action of the protein p66^shc^, which oxidates cytochrome c and partially reduces molecular oxygen to O_2_•^−^ [82], in the matrix by metabolic enzymes (aconitase and α ketoglutarate dehydrogenase) or in the outer mitochondrial membrane by the monoamine oxidases (MAO A and MAO B) [94]. Of note, a little amount of O_2_•^−^ may translocate in the cytosol through the voltage-dependent anion channel (VDAC) in the outer mitochondrial membrane, while the majority is dismutated into H_2_O_2_ by mitochondrial SOD (Mn-SOD or SOD2), which, in turn, may diffuse in the cytosol through mitochondrial membranes [52,95]. However, H_2_O_2_ levels must be tightly regulated to avoid cytotoxic effects (protein and lipid modification, DNA damage, programmed cell death) and H_2_O_2_ may be converted into H_2_O by catalase, glutathione peroxidase, and peroxiredoxins [52,93].

### 2.5. Arachidonic-Acid-Metabolizing Enzymes

Arachidonic acid is a conditionally essential polyunsaturated fatty acid that, in endothelial cells, plays a crucial role in regulating NO release and angiogenesis [43,96,97]. Arachidonic acid is cleaved by glycerophospholipids on the plasma membrane or the nuclear envelope by phospholipase A2 (PLA2), PLC, and phospholipase D (PLD) (Figure 1) [98] and may be metabolized into an impressive array of bioactive eicosanoids, e.g., prostanoids, thromboxane, leukotrienes, and epoxyeicosatrienoic acids (EETs) (Figure 1), by three distinct families of enzymes, respectively: COXs, LOXs, and CYP ω-hydroxylases and epoxygenases [98,99]. ROS may be generated as byproducts of arachidonic acid oxidation by several COX (e.g., COX-1), LOX (e.g., 5-LOX) and CYP (e.g., CYP2C8 and 9) enzymes [98,99,100,101]. Intriguingly, LOXs- and COXs-derived arachidonic acid metabolites may stimulate multiple NOX isoforms, including NOX1 and NOX4, to induce ROS signaling in response to chemical stimulation [98,99].

### 2.6. ROS Elimination

Endothelial cells have developed a sophisticated antioxidant defense system to prevent intracellular ROS accumulation and endothelial dysfunction, including glutathione (GSH), SOD, catalase, peroxiredoxins (Prx), and thioredoxin (Trx) [46,82]. Briefly, GSH is central to balancing the cellular redox state, and the ratio of the reduced GSH to oxidized disulfide GSH (GSH/GSSG) is regarded as a reliable indicator of oxidant stress. S-glutathionylation can interfere with the irreversible modifications of protein thiol groups by H_2_O_2_ and thus maintains correct redox signaling and prevents cellular damage. The exchange between GSH and GSSG is regulated by GSH peroxidase (GPx), which catalyzes the oxidation of GSH to GSSG, and by the NADPH-dependent GSH reductase, which mediates the reduction of GSSG to GSH [102]. SOD, in turn, represents the main endothelial enzymatic control system of O_2_•^−^ and, in mammalian cells, exists in three isoforms: cytoplasmic SOD (SOD-1 or Cu/Zn-SOD), mitochondrial SOD (SOD-2 or Mn-SOD), and extracellular SOD (SOD-3 or EC SOD). O_2_•^−^ is quicky dismutated by SOD-1 and SOD-2 into the less reactive H_2_O_2_, which is subsequently reduced to water and O_2_ by catalase or to water and oxidized glutathione by GPx. Finally, the Trx system consists of a family of 12 kDa oxidoreductases that maintain the thiol groups of reduced Prx in the reduced state, thereby maintaining Prx-dependent reduction of H_2_O_2_ to water. Of note, the majority of these antioxidant enzymatic systems impinge on NADPH as the ultimate donor of reductive power [82,103].

## 3. ROS Evoke or Modulate Intracellular Ca^2+^ Release in Endothelial Cells

The endothelial Ca^2+^ response to extracellular stimuli is usually triggered by endogenous Ca^2+^ mobilization and then sustained over time by store- or second messengers-operated Ca^2+^-permeable channels belonging to the TRP superfamily [5,6,15,26]. The endoplasmic reticulum (ER) represents the largest endothelial Ca^2+^ store by containing ≈75% of the intracellular Ca^2+^ reservoir [104] by virtue of the high Ca^2+^ affinity of SERCA2B, which mainly accounts for ER Ca^2+^ recharging [105]. InsP_3_Rs provide the main pathway for ER Ca^2+^ release upon stimulation of either G_q/11_PCRs or TKRs on the plasma membrane [26,106]. Endothelial G_q/11_PCRs recruit phospholipase Cβ2 (PLCβ2) or PLCβ3 to cleave the plasma membrane lipid phosphatidylinositol 4,5-bisphosphate (PIP_2_) into diacylglycerol (DAG) and InsP_3_, which, in turn, diffuses toward ER cisternae to gate InsP_3_Rs and mobilize ER Ca^2+^ into the cytosol [26]. PLCγ1 couples TKRs to InsP_3_ production and InsP_3_-dependent signaling in the endothelial lineage [107]. All three InsP_3_R isoforms, i.e., InsP_3_R1–3, are present in endothelial cells [108,109,110], whereas only InsP_3_R3 is absent in circulating ECFCs [111]. Intriguingly, InsP_3_Rs require a permissive Ca^2+^ concentration (50-200 nM) in the surrounding microenvironment to be engaged by the InsP_3_ produced in response to extracellular stimulation [112]. In addition, InsP_3_R1 channel activity is tightly sensitive to the cellular redox state [62]; physiologically relevant ROS may result in the oxidation of critical endogenous thiol residues and sensitize InsP_3_Rs either to the low ambient InsP_3_ concentration [113,114] or to resting [Ca^2+^]_i_ [115,116]. Furthermore, InsP_3_R channel activity in vascular endothelial cells may also be modulated by mitochondria, which may establish close contacts with ER cisternae (known as mitochondria-associated ER membranes or MAMs) [117] and inhibit InsP_3_-induced Ca^2+^ release in endothelial cells in a H_2_O_2_-dependent manner [118]. Ryanodine receptors (RyRs) provide as an alternative pathway to release intraluminal Ca^2+^ either through the process of Ca^2+^-induced Ca^2+^ release (CICR) [119,120] or upon binding of the Ca^2+^-releasing second messenger, cyclic ADP ribose (cADPr) [121]. As reviewed elsewhere [26,106], endothelial RyRs are not as widely distributed as InsP_3_Rs across peripheral vasculature and are absent in circulating ECFCs [122]. Therefore, RyRs play a minor role in the onset and propagation of intracellular Ca^2+^ waves in the endothelial lineage. Finally, growing evidence has convincingly shown that the acidic vesicles of the endolysosomal (EL) system provide an additional Ca^2+^ reservoir that can be exploited by extracellular stimuli to increase the endothelial [Ca^2+^]_i_ [123]. The EL Ca^2+^ pool may be discharged by the Ca^2+^-releasing second messenger, nicotinic acid adenine dinucleotide phosphate (NAADP) via TPCs, of which two isoforms are present in endothelial cells, i.e., TPC1 and TPC2 [109,123], whereas ECFCs only express TPC1 [27]. In accord with the so-called “trigger hypothesis” [124,125,126], NAADP-induced EL Ca^2+^ release via TPCs may deliver the permissive Ca^2+^ pulse required by InsP_3_Rs to mediate ER Ca^2+^ mobilization upon priming by InsP_3_ also in the endothelial lineage [10,27].

In the following Sections, we focus on the wide literature supporting the notion that ROS stimulate InsP_3_R channel activity and that H_2_O_2_ also controls SERCA-mediated ER Ca^2+^ sequestration.

### 3.1. Superoxide Anion, O_2_•^−^, and Hydroxyl Radical, OH•, Evoke Intracellular Ca^2+^ Release in Vascular Endothelial Cells

A flurry of investigations mainly carried out during the last decade of the twentieth century demonstrated that ROS were able to increase the endothelial [Ca^2+^]_i_ (Table 1). As nicely reviewed in [127], oxidant signaling was investigated by challenging endothelial cells with the O_2_•^−^-generating systems, (xypo)xanthine (H)X/XO [128,129], the H_2_O_2_-generating system, glucose/glucose oxidase (G/GO) [79,129], with exogenous H_2_O_2_ [130,131], with diamide [115,116], with thimerosal [132], or with tert-butyl hydroperoxide (t-BOOH) [133,134]. High doses of HX/XO caused an increase in endothelial [Ca^2+^]_i_ resulting from InsP_3_-induced ER Ca^2+^ release and extracellular Ca^2+^ entry (Table 1) [135]. This Ca^2+^ signal was attenuated by scavenging O_2_•^−^ with SOD and by preventing OH• formation through the Fenton reaction, whereas the residual increase in [Ca^2+^]_i_ observed in the presence of SOD was removed by scavenging H_2_O_2_ with catalase [79]. As more widely discussed below, OH•-induced peroxidation of membrane lipids may promote Ca^2+^ influx through TRP Ankyrin 1 (TRPA1) in vascular endothelial cells [77]. Subsequent reports showed that the intracellular generation of lower doses of O_2_•^−^ could either sensitize InsP_3_Rs to mobilize ER Ca^2+^ and thereby engage the SOCE pathway in response to agonist stimulation [136] or evoke an increase in [Ca^2+^]_i_ (Table 1) [137,138]. Hajnóczky’s group recently demonstrated that exogenous O_2_•^−^ has the potential to oxidize multiple thiol groups within InsP_3_R1 and InsP_3_R2 channel proteins, thereby sensitizing InsP_3_Rs to mediate ER Ca^2+^ release [114]. The mechanisms whereby oxidant signaling could promote InsP_3_-induced ER Ca^2+^ mobilization are described in Section 3.2.

### 3.2. H_2_O_2_ Triggers InsP_3_-Induced ER Ca^2+^ Release in Vascular Endothelial Cells

The notion that H_2_O_2_ could serve as a Ca^2+^ releasing second messenger in vascular endothelial cells was originally suggested by the inhibitory effect exerted by catalase on the Ca^2+^ response to (H)X/XO (Table 1) [79,127,129]. The first clear-cut characterization of H_2_O_2_-induced spatiotemporal endothelial Ca^2+^ signals was provided by Ziegelstein’s group (Table 1) [139]. In their first landmark paper [139], Ziegelstein’s group detailed how exogenous delivery of H_2_O_2_ induced a dose-dependent increase in [Ca^2+^]_i_ in human aortic endothelial cells (HAECs). At concentrations ≥100 µM, H_2_O_2_ induced repetitive oscillations in [Ca^2+^]_i_, which overlapped a gradual elevation in [Ca^2+^]_i_ and then merged into a sustained plateau phase [139]. H_2_O_2_-induced intracellular Ca^2+^ oscillations were independent of extracellular Ca^2+^ entry but disappeared upon depletion of the InsP_3_-sensitive ER Ca^2+^ pool [139]. Upon stimulation with high (>1 mM) doses of H_2_O_2_, the intracellular Ca^2+^ oscillations accelerated and immediately fused in a prolonged plateau that maintained the [Ca^2+^]_i_ well above prestimulation levels [139]. Two independent investigations confirmed that H_2_O_2_ caused a massive reduction in ER Ca^2+^ concentration ([Ca^2+^]_ER_) following InsP_3_R stimulation in human umbilical vein endothelial cells (HUVECs) [140] and calf pulmonary artery endothelial cells (CPAECs) (Table 1) [128]. This might explain why prolonged exposure (1 h) to peroxides may inhibit the subsequent endothelial Ca^2+^ response to extracellular stimulation [134]. H_2_O_2_ could induce InsP_3_-dependent Ca^2+^ release from the ER by directly engaging PLCγ1 [141,142] and/or by stimulating InsP_3_Rs [62,113,114]. Exogenous delivery of intermediate to high doses (500 µM-5 mM) of H_2_O_2_ promoted InsP_3_ production in mouse aortic and mesenteric artery endothelial cells [138], whereas it is still unclear whether lower concentrations of this peroxide stimulate PIP_2_ hydrolysis, as reported in other cell types [141,142]. Alternately, changes in the thiol redox state could prime InsP_3_R1 to be activated either by the low ambient InsP_3_ concentration [113,114] or by resting [Ca^2+^]_i_ [115,116]. Although InsP_3_R2 and InsP_3_R3 may undergo H_2_O_2_-dependent sulfhydryl redox modifications [143], a preliminary characterization of the functional roles and reactivity of cysteine residues is available only for InsP_3_R1. The primary sequence of InsP_3_R1 presents 60 thiol groups and, of these, ≈70% are sensitive to oxidant-induced post-translational changes [144]. A recent report by Hajnoczky’s group revealed that two specific cytosolic (Cys-292 and Cys-1415) and two intraluminal (Cys-2496 and Cys-2533) cysteine residues of InsP_3_R1 are oxidized under basal conditions in intact cells, whereas H_2_O_2_ may oxidize three additional cysteines (Cys-206, Cys-214, and Cys-1397) that are clustered within the NH_2_ terminal domain [113]. Oxidative modifications of RyRs have been extensively investigated and include disulfide crosslinking (inter-/intramolecular covalent bondage of two free thiols) and S-glutathionylation (i.e., incorporation of GSH into a cysteine thiol) [145]. Disulfide bridge formation has been reported only within the third lumenal loop of the InsP_3_R1 protein [146]. The ER is the organelle showing the highest intraluminal H_2_O_2_ levels [147] and, therefore, oxidant stress is unlikely to target InsP_3_R1 by inducing intramolecular disulfide bonds [114]. However, Schilling’s group reported that H_2_O_2_ and diamide, a membrane-permeable thiol-oxidizing compound, induced intracellular Ca^2+^ oscillations in cultured endothelial cells by priming InsP_3_R1 to CICR via S-glutathionylation of the third lumenal loop [115,116]. According to the proposed model, a decrease in the ER redox state induced by oxidant signal uncouples the ER resident protein, Erp44, from the free cysteines present in the loop, thereby increasing InsP_3_R channel activity [115,146]. Interestingly, Erp44 is associated to InsP_3_R1 but not to InsP_3_R2 and InsP_3_R3, and this physical interaction is regulated by lumenal redox state, Ca^2+^, and pH [146]. It is still unclear whether the redox potential (around -200 mV) is homogenous or varies among different ER domains [148], while there is no doubt that the [Ca^2+^]_ER_ presents intraluminal gradients [149]. Therefore, the different pattern of InsP_3_ expression (InsP_3_R1 vs. InsP_3_R2 and InsP_3_R3) and/or inhomogeneities in local luminal Ca^2+^ levels could add a further layer of complexity to H_2_O_2_-dependent regulation of endothelial InsP_3_Rs. For instance, depending on the ER redox state, the same oxidant stress could be more effective at eliciting intracellular Ca^2+^ signals in endothelial cells from some vascular beds of a given species (e.g., those with a lower ER redox potential) but not in others (e.g., those with a higher ER redox potential), as reported in [115,138,150]. Furthermore, although sometimes unable to increase the endothelial [Ca^2+^]_i_, acute oxidant signaling via either H_2_O_2_ [150] or O_2_•^−^ [136] could sensitize the subsequent Ca^2+^ response to InsP_3_-producing autacoids (Table 1). These observations concur with the hypothesis that it is the local microenvironment (e.g., higher or lower [InsP_3_]) around InsP_3_Rs that dictates their ROS sensitivity. Additional mechanisms that may underlie the differential effects of H_2_O_2_ and O_2_•^−^ on the endothelial Ca^2+^ toolkit could depend on the vascular bed [127,138], on the accessibility of the reactive thiols [62,114], on redox compartmentalization [148], or on the physical interaction of InsP_3_Rs with auxiliary proteins, e.g., homer-1, which serve as additional sensors of oxidant stress [151].

**Table 1:** Representative studies showing the effect of ROS on endothelial Ca^2+^ homeostasis.

ROS	Mechanism of ROS Stimulation	Dose of ROS or of ROS-Generating Enzymes	ROS Scavenger	Endothelial Cell Type	Effect on Intracellular Ca^2+^ Homeostasis	Reference
H_2_O_2_	Acute exposure	1-5-10 mM	Not used	CJVECs	ICR and ECI	[130]
H_2_O_2_	Acute exposure	100 µM	Not used	CPAECs	ICR and ECI	[131]
H_2_O_2_	Acute exposure	500 µM	Not used	SRLECs HUVECs	ICR	[129]
H_2_O_2_	Acute exposure	100 µM-10 mM	Not used	HAECs	ICR	[139]
H_2_O_2_	Acute exposure	10 µM	Not used	HUVECs	Not determined	[79]
H_2_O_2_	Acute exposure	1 mM	Not used		ICR	[140]
H_2_O_2_	Acute exposure	5 mM	Cat, effect DMSO, no effect	MAECs MesAECs	ICI and ECI	[138]
H_2_O_2_	Acute exposure	100 µM		BAECs	ICI	[115]
H_2_O_2_	Acute exposure	10-100 µM	Cat, effect NAC, effect	HUVECs	Increases agonists-induced Ca^2+^ signaling	[150]
H_2_O_2_	HX/XO	1 mM HX/2 mU/mL XO	Cat, effect SOD, no effect O-phen, no effect	SRLECs HUVECs	ICR	[129]
H_2_O_2_	G/GO	10 mM G/2 mU/mL GO	Cat, effect SOD, no effect O-phen, no effect	SRLECs HUVECs	ICR	[129]
H_2_O_2_	HX/XO	0.5 mM HX/50 mU/mL XO	Cat, effect SOD, no effect	CPAECs	ICR and ECI	[128]
H_2_O_2_	G/GO	10 nM G/[GO] →10 nM H_2_O_2_/_mL_/min	Not used	HUVECs	Not determined	[79]
H_2_O_2_, O_2_•^−^ and ^•^OH	HX/XO	2 mM HX/[XO] → O_2_^-^ nM/mL/min	Cat, effect SOD, effect O-phen, effect	HUVECs	ICI and ECI	[79,135]
H_2_O_2_ and O_2_•^−^	HX/XO	200 µM HX/20 mU/mL XO	Cat, effect SOD, effect	MAECs MesAECs	ICI and ECI	[138]
O_2_•^−^	HX/XO	1 mM HX/150 mU/mL XO	SOD, effect	PAECs	Increases agonist-induced ICI and SOCE	[136]
H_2_O_2_, O_2_•^−^ and ^•^OH	X/XO	200 µM HX/2 mU/mL XO	Cat, effect SOD, effect O-phen and Def, effect	PAECs	ICI and ECI	[137]

Abbreviations: BAECs: bovine aortic endothelial cells; CJVECs: canine jugular venous endothelial cells; CPAECs: calf pulmonary artery endothelial cells; Def: deferoxamine: DMSO: dimethyl sulfoxide; G/GO: glucose/glucose oxidase; HAECs: human aortic endothelial cells; ICR: intracellular Ca^2+^ release; ECI: extracellular Ca^2+^ influx; MAECs: mouse aortic endothelial cells; MesAECs: mesenteric artery endothelial cells; NAC: N-acetylcysteine; O-phenanthroline: O-phen; PAECs: porcine aortic endothelial cells; SRLECs: sinusoidal rat liver endothelial; X/XO: xanthine/xanthine oxidase. Def, DMSO and O-phen prevent ^•^OH formation by inhibiting the Fentom reaction.

### 3.3. Evidence That ROS May Trigger Agonists-Induced Intracellular Ca^2+^ Release in Vascular Endothelial Cells

Intracellular ROS can be produced upon recruitment of G_q/11_PCRs on the plasma membrane and thereby contribute to shape endothelial Ca^2+^ signals. Early work by Ziegelstein’s group revealed that the activation of endothelial NOX by exogenous NADPH resulted in the generation of H_2_O_2_ and O_2_•^−^, thereby increasing InsP_3_R sensitivity to ambient [InsP_3_] and promoting InsP_3_-induced ER Ca^2+^ mobilization [152]. Subsequently, the same group showed that NOX sustained the intracellular Ca^2+^ oscillations evoked in HAECs by histamine [153], an inflammatory mediator that exploits intracellular Ca^2+^ signaling to reduce endothelial permeability and facilitate leukocyte transendothelial migration [154]. A recent investigation confirmed that NOX was also be involved in histamine-induced increase in [Ca^2+^]_i_ and von Willebrand factor (vWF) secretion in HUVECs [50]. These authors suggested that, in addition to InsP_3_Rs, lysosomal TPCs contribute to H_2_O_2_-induced intracellular Ca^2+^ mobilization downstream NOX engagement [155]. However, several issues remain to be clarified. First, which NOX isoform triggers histamine-induced Ca^2+^ signaling in vascular endothelial cells? Second, does NOX initiate the endothelial Ca^2+^ response arising downstream of other G_q/11_PCRs? Third, which ROS are generated downstream NOX activation to give raise to endothelial Ca^2+^ signals? Answering these questions is crucial to delineate the mechanisms whereby ROS exploit endothelial Ca^2+^ signaling to regulate vascular functions. NOX is not the only enzyme driving ROS production during the early phases of an endothelial Ca^2+^ signal. An elegant study revealed that muscarinic M2 receptors may activate cytosolic PLA2 (cPLA2) in the endothelial monolayer covering rat mesenteric arteries, thereby promoting H_2_O_2_ generation upon CYP450 2C9 isoform-mediated metabolism of AA [156]. The hydroxyl radical, •OH, may then be produced from H_2_O_2_ to sensitize InsP_3_Rs to mediate intracellular Ca^2+^ release and Ca^2+^-dependent vasodilation via NO release and EDH [156]. Alternately, acetylcholine was found to impinge on CYP450 2C11 and CYP450 2C23 isoforms to induce H_2_O_2_ production and stimulate EDH in rat renal arteries [157].

Intriguingly, a number of autacoids may induce endothelial ROS release through an increase in [Ca^2+^]_i_ that results in the activation of the Ca^2+^/CaM-sensitive NOX5 isoform. For instance, bradykinin-dependent ROS production in PAECs requires InsP_3_-dependent ER Ca^2+^ release, whereas SOCE is ineffective at engaging NOX5 [64]. Similarly, angiotensin II and endothelin 1 promote O_2_•^−^ production in HMECs in a Ca^2+^/CaM-dependent manner, but the underlying signaling pathway has not been deciphered [158]. Future work should assess whether ROS produced upon an initial elevation sustain Ca^2+^ signaling over time through the subsequent activation of ROS-sensitive Ca^2+^-permeable channels on the ER and/or the plasma membrane.

### 3.4. Evidence That ROS Can Modulate SERCA2B Activity during Agonists-Induced Ca^2+^ Signals in Vascular Endothelial Cells

SERCA activity finely shapes the intracellular Ca^2+^ waveforms evoked by prolonged stimulation in cultured endothelial cells by reloading the ER with Ca^2+^, thereby setting up the onset of the next Ca^2+^ spike [10,11,16]. As recently reviewed in [26], SERCA2B is the main responsible for ER Ca^2+^ refilling in vascular endothelium. SERCA2 presents a cysteine residue in the cytosolic P-domain (Cys674) and a pair of cysteine thiols (Cys875 and Cys887) in the longest intralumenal loop 4 (L4) [159]. It has been shown that S-glutathionylation of Cys674 increases SERCA2B Ca^2+^ pumping activity in the cardiovascular system [160,161]. Conversely, the irreversible oxidation of Cys674 prevents S-glutathionylation and inhibits SERCA2B activity [162,163]. An early report demonstrated that NO-induced S-glutathionylation at Cys674 enhanced VEGF-induced ER Ca^2+^ release through RyRs and SOCE activation in HAECs, thereby supporting endothelial cell migration [105]. The same group showed that VEGF-induced SOCE and endothelial cell migration are driven by S-glutathionylation of SERCA2B Cys674 by NOX4-produced H_2_O_2_, although ROS signaling is then maintained by NOX2 [164]. These observations demonstrate that the endothelial ER senses ROS to either recharge its Ca^2+^ content (via SERCA2B) or to release intraluminal Ca^2+^ (mainly via InsP_3_Rs). This would prevent the depletion of ER Ca^2+^ content during physiological redox signaling, a virtuous goal that can be further achieved through ROS-dependent SOCE activation (see below). ROS sensitivity of SERCA2B Cys674 is also relevant to vascular regrowth upon an ischemic insult. VEGF-induced ER Ca^2+^ release, migration, and tube formation were impaired in hypoxic endothelial cells isolated from a transgenic mouse lacking half of the redox-sensitive thiol groups at Cys674 [165]. In the same animal model, blood flow recovery after hindlimb ischemia was severely impaired, which is consistent with the scarce activation of angiogenic activity within the injured tissue [165]. A follow-up study showed that, when the reversible S-glutathionylation of SERCA2B is compromised, the endothelial expression of ER oxidoreductin-1α (ERO1) is impaired, which further reduces the angiogenic response to hypoxic conditions due to the increased ER stress [165].

## 4. ROS Modulate Store-Operated Ca^2+^ Entry in Vascular Endothelial Cells

SOCE represents a ubiquitous pathway for extracellular Ca^2+^ entry in endothelial cells across the whole peripheral vasculature [26,166,167]. Endothelial SOCE is engaged by the InsP_3_-dependent depletion of the ER Ca^2+^ store by chemical cues, such as growth factors, hormones, and autacoids, to refill the ER with Ca^2+^, prolong the increase in [Ca^2+^]_i_ over time, and recruit a plethora of Ca^2+^-dependent decoders. Thus, SOCE regulates most of endothelial functions, ranging from NO release and vWF secretion to the control of endothelial permeability and proliferation [9,26,166,167,168]. Similarly, SOCE is crucial to ensure proper intracellular Ca^2+^ signaling in circulating ECFCs recruited to ischemic tissues to participate in vascular regrowth [97,111,169]. The molecular makeup of endothelial SOCE may change depending on the vascular bed, but briefly addressing this controversial issue is necessary to understand how redox signaling regulates agonist-evoked extracellular Ca^2+^ entry in the endothelial lineage. Three independent studies reported that SOCE is mediated by the physical interaction between stromal interaction molecule 1 (STIM1) and Orai1 channels in HUVECs [170,171,172], the most widespread endothelial cell model. As extensively reviewed elsewhere [166,167,168], STIM1 is a single-pass transmembrane dimeric protein that serves as a sensor of [Ca^2+^]_ER_ due to its low affinity for Ca^2+^ (≈200 µM). STIM1 is activated by a large reduction in [Ca^2+^]_ER_ and is thereafter prompted to undergo a conformational remodeling and translocate to close (10-20 nm) junctions between ER and plasma membrane, known as puncta. Herein, STIM1 physically interacts with and gates Orai1, which provides the pore forming subunit of a store-operated channel termed the Ca^2+^ release-activated Ca^2+^ (CRAC) channel. STIM1 and Orai1 were also shown to mediate SOCE in HAECs [105,173], in human pulmonary artery endothelial cells (HPAECs) [170], and in the HUVEC-derived endothelial cell line, EA.hy926 [174,175]. Vascular endothelial cells also express the STIM1 and Orai1 paralogues, i.e., STIM2, Orai2, and Orai3 [109,110,173]. STIM2, which is a weaker activator of Orai1 and displays a higher affinity for intraluminal Ca^2+^ (≈500 µM), is activated upon a milder depletion of the ER Ca^2+^ store and, therefore, stimulates Orai1 to mediate constitutive Ca^2+^ entry in HUVECs [176]. It has been suggested that STIM2 recruits STIM1 at ER–plasma membrane junctions to engage Orai1 at low agonist concentration [177], whereas STIM2 contribution to SOCE decreases as agonist concentration decreases [178]. Whether this interaction between STIM paralogues also occurs in endothelial cells is still unknown. Orai2 and Orai3, in turn, may serve as dominant negative of Orai1-mediated Ca^2+^ entry [179,180]. A recent series of investigations by Trebak’s group confirmed that the distinct Orai isoforms may assemble to form naive CRAC channels, although the precise stoichiometry of Orai heteromers is likely to be cell-specific [178,181]. While the role of Orai3 in endothelial I_CRAC_ and SOCE has never been clearly addressed, Orai2 serves as a negative regulator of Orai1-mediated Ca^2+^ entry in bovine brain capillary endothelial cells [182]. Understanding which STIM and Orai isoforms contribute to endothelial SOCE is relevant to ROS signaling, which may differentially affect STIM1 vs. STIM2 [183,184] as well as Orai1 vs. Orai3 [185], as is more widely discussed in Section 4.1.

### 4.1. H_2_O_2_ Modulates STIM and Orai Proteins: Direct and Indirect Mechanisms

STIM and Orai proteins present a variable number of reactive cysteines that impart redox sensitivity to SOCE. We refer the readers to a couple of review articles in which the mechanisms and functional consequences of STIM and Orai modulation by the redox state were extensively described [184,186]. Briefly, STIM1 displays two highly conserved thiol groups (Cys49 and Cys56) in the intraluminal NH_2_ terminal tail, which are in close proximity to the Ca^2+^-binding site and are responsible for STIM1 regulation by ROS. H_2_O_2_-dependent S-glutathionylation of Cys49 and Cys56 decreases STIM1 affinity for Ca^2+^, thereby mimicking the effect of ER Ca^2+^ depletion and promoting STIM1 activation and translocation to the plasma membrane [187]. Conversely, the intraluminal protein, Erp57, could promote the formation of a disulfide bridge between Cys49 and Cys59 that prevents STIM1 activation and recruitment into submembrane puncta upon a reduction in [Ca^2+^]_ER_ [188]. Although some discrepancies between these two studies have been highlighted [184,186], the redox-dependent S-glutathionylation of Cys49 and Cys56 could release STIM1 from Erp57-dependent inhibition and result in SOCE activation. STIM2 protein presents a higher number of cysteine residues as related to STIM1 (15 vs. 4), and most of these (11 vs. 1) are located in the cytosolic COOH-terminal domain [184,186], which underlies STIM oligomerization and gating of Orai channels [189]. A recent investigation showed that H_2_O_2_-dependent sulfonylation of the cytoplasmic Cys313 hinders STIM2 oligomerization and, therefore, prevents Orai1 activation [183]. On the plasma membrane, Orai channels consist of homo- and heteroexamers [178,181], in which each subunit presents four transmembrane (TM) domains with intracellularly located NH_2_- and COOH-terminal tails [189]. Orai1 and Orai2 share three highly conserved cysteine residues: Cys126 in the second TM domain, Cys143 in the cytosolic loop connecting the second and third TM domains, and Cys195 at the extracellular end of the third TM domain. Orai3 lacks Cys195 but contains two additional cysteine residues in the long extracellular loop connecting the third and fourth TM domains [184,186]. Bogeski et al. unveiled that Cys195 represents the major reactive cysteine of Orai1 and is responsible for H_2_O_2_-dependent inhibition of I_CRAC_ and SOCE in HEK293 cells transfected with STIM1 and Orai1, Jurkat T cells, and CD4^+^ T cells [185,186]. Cys195 oxidation interferes with Orai1 subunit interaction and prevents effective Orai1 gating by STIM1, thereby locking the CRAC channel in a closed conformation [190]. Conversely, Orai3, which lacks the extracellular Cys195 that renders CRAC channels sensitive to oxidative microenvironment, is redox-insensitive [185]. Intriguingly, the insertion of Orai3 in the heteromeric complex responsible for SOCE renders Orai1 less sensitive to oxidative stress, as reported in effector T_H_ cells [185] and prostate cancer cells [191].

Besides direct modification of reactive thiols within STIM and Orai proteins, ROS signaling could indirectly modulate the I_CRAC_ by targeting InsP_3_Rs. For instance, Grupe et al. provided the evidence that H_2_O_2_ triggers InsP_3_-mediated ER Ca^2+^ release to activate SOCE in RBL-2H3 cells, HEK293 cells and Jurkat T cells [192]. The same signaling pathway was responsible for H_2_O_2_-induced SOCE channels in rat coronary artery vascular smooth muscle cells [193] and, probably, human keratinocytes [194]. An alternative, and intriguing, mode of indirect SOCE activation by ROS signaling could impinge on the S-glutathionylation of SERCA2B Cys674. Indeed, an increase in the rate of ER Ca^2+^ refilling by SERCA2B would lead to ER Ca^2+^ overload, which, in turn, is able to stimulate InsP_3_Rs and thereby initiate the function cross-talk between STIM and Orai proteins [195]. Paradoxically, SERCA2B inhibition by excessive production of oxidants could lead to SOCE activation as intraluminal Ca^2+^ efflux through ER leakage channels is no longer counteracted by SERCA2B-mediated sequestration into ER lumen and may lead to ER Ca^2+^ depletion [33,196].

### 4.2. Evidence That ROS May Modulate SOCE in Vascular Endothelial Cells

Early reports showed that acute generation of intracellular ROS induces Ca^2+^ influx in endothelial cells from multiple vascular beds (Table 1), including HUVECs and SRLECs [129], CJVECs [130], CPAECs [128,131], MAECs and MesAECs [138], and PAECs [136,137]. These insightful investigations mainly focused on the ROS species and/or the source (intracellular vs. extracellular) of the Ca^2+^ response. These studies hinted at InsP_3_Rs as the main ER Ca^2+^-releasing channel activated by ROS [115,116,128,138,139,140], as pointed out in Section 3.2. Conversely, there was not any straightforward conclusion on the molecular nature of the ROS-sensitive Ca^2+^ entry pathway in the plasma membrane. It is worth of recalling that these investigations were carried out in the pre-TRP channel era and that, in those pioneering days, SOCE was regarded as the most important Ca^2+^ entry pathway in vascular endothelial cells [127]. Indeed, based upon the findings that H_2_O_2_-induced Ca^2+^ entry was associated to H_2_O_2_-induced depletion of the InsP_3_-sensitive ER Ca^2+^ pool (Table 1), many authors drew the reasonable conclusion that the acute exposure of vascular endothelial cells to H_2_O_2_ indirectly led to SOCE activation, i.e., upon InsP_3_-induced ER Ca^2+^ depletion [129,130,131]. A more recent report confirmed that platelet lysate induced NOX4 activation in the mouse brain immortalized cell line, bEND5, thereby promoting InsP_3_-induced ER Ca^2+^ release and SOCE [197]. Subsequently, the same group reported that H_2_O_2_ released by buckwheat honey triggers InsP_3_-induced ER Ca^2+^ release followed by extracellular Ca^2+^ entry in the same cell line [198]. Honey-evoked Ca^2+^ influx was sensitive to econazole, an imidazole derivative that has long been known to affect SOCE [199]. Furthermore, SOCE has been established as the main responsible for prolonged Ca^2+^ entry in bEND5 cells in response to chemical stimulation [11,24,110,197]. Thus, although gene silencing of STIM and/or Orai proteins is required to confirm this hypothesis, SOCE is likely to sustain H_2_O_2_-induced Ca^2+^ entry in bEND5 cells.

### 4.3. Prolonged Exposure to Oxidant Stress Impairs SOCE in Vascular Endothelial Cells

While the clear-cut evidence that acute addition to ROS leads to SOCE activation is still missing, there is a large agreement upon SOCE inhibition following a prolonged exposure to oxidant stress in vascular endothelial cells [8,166]. Early work showed that 1 h incubation of CPAECs with t-BOOH, which is metabolized by GPx and, therefore, causes a reduction in the endogenous antioxidant system, remarkably reduced SOCE, although it did not affect the InsP_3_-sensitive ER Ca^2+^ pool [134]. This observation was later confirmed by Blatter’s group [200] and suggests that either the store-operated channel on the plasma membrane or the [Ca^2+^]_ER_-sensing mechanism are altered by this treatment. A more recent investigation showed that incubation of the bovine brain cerebrovascular endothelial cells with H_2_O_2_ (30 µM) for 24 h remarkably inhibited SOCE, probably via oxidation of the extracellular Cys195 in the third TM domain of Orai1 [201]. Intriguingly, longer (>24 h) exposure to intracellular ROS could result in a significant upregulation of endothelial STIM1 and Orai1 proteins. Tamareille et al. described that culturing HUVECs for 96 h in the presence of high glucose (HG) (30 mM) resulted in a dramatic increase in the magnitude of both I_CRAC_ and SOCE that was dependent, at least partially, on intracellular H_2_O_2_ generation [202]. These authors suggested that prolonged oxidant stress promote the upregulation of the molecular components of SOCE, i.e., STIM1 and Orai1 in HUVECs [170,171,172,203], through the recruitment of the Ca^2+^-dependent phosphatase, calcineurin [202]. In agreement with this observation, Daskoulidou et al. found that chronic treatment (72 h) with HG (25 mM) stimulated the Ca^2+^-dependent effector, calcineurin, to promote the nuclear translocation of nuclear factor of activated T cells 3 (NFATc3), thereby increasing the protein expression of Orai1–3 and STIM1–2 in multiple types of human endothelial cells [173]. These authors proposed that the overproduction of ROS, mainly H_2_O_2_, under the oxidant conditions imposed by HG could lead to an increase in endothelial [Ca^2+^]_i_ by activating InsP_3_Rs and/or SOCE [173]. This mechanism, although plausible, remains to be demonstrated and deserves further attention because of the pathological implications of prolonged oxidant stress, as is more extensively described in Section 6.

## 5. ROS Mediate Extracellular Ca^2+^ Influx through the Activation of Transient Receptor Potential (TRP) Channels

The TRP superfamily of nonselective cation channels comprise 28 isoforms subdivided in six subfamilies according to their sequence homology: TRP canonical (TRPC1-7), TRP vanilloid (TRPV1-6), TRP melastatin (TRPM1-8), TRPA1, TRP mucolipin (TRPML1-3), and TRP polycystin (TRPP) [5,15,204]. TRP channels are featured by six TM (TM1-6) α-helix segments, with cytosolic NH_2_- and COOH-termini, and they assemble into a tetrameric complex around the reentrant pore loop between TM5 and TM6 of each subunit [5,204]. The NH_2_ and COOH termini present a wide variability in length and function in different TRP subfamilies, may interact with regulatory proteins, cytoskeletal structures, or Ca^2+^ sensors, such as STIM1 and calmodulin (CaM). Furthermore, the COOH terminus of TRPM2, TRPM6, and TRPM7 present an enzymatic domain that is involved in channel gating and downstream intracellular signaling pathways [204,205]. Although they are similar to voltage-gated K^+^ channels, TRP channels lack the voltage sensor in TM4 [205]. TRP channels are permeable to monovalent (i.e., Na^+^ and Ca^2+^) and divalent (i.e., Ca^2+^ and Mg^2+^) cations, but they have different relative permeability to Ca^2+^ and Na^+^ (P_Ca_/P_Na_). For instance, TRPM4 and TRPM5 are almost impermeable to Ca^2+^ (P_Ca_/P_Na_ < 0.01), whereas TRPV1, TRPV4, and TRPA1 present a high Ca^2+^ permeability (P_Ca_/P_Na_~6-10) [204,205]. Endothelial TRP channels regulate a plethora of vascular functions, including vascular tone, endothelial permeability, and angiogenesis, and most of them are recognized as polymodal (i.e., activated by multiple chemical and physical cues) routes for extracellular Ca^2+^ entry [3,5,15]. A number of TRP isoforms may also serve as redox sensors and contribute to regulate ROS-dependent endothelial functions.

### 5.1. TRPC3 and TRPC4 Form a Redox-Sensitive Ca^2+^-Permeable Channel in Vascular Endothelial Cells

**Figure 2:**
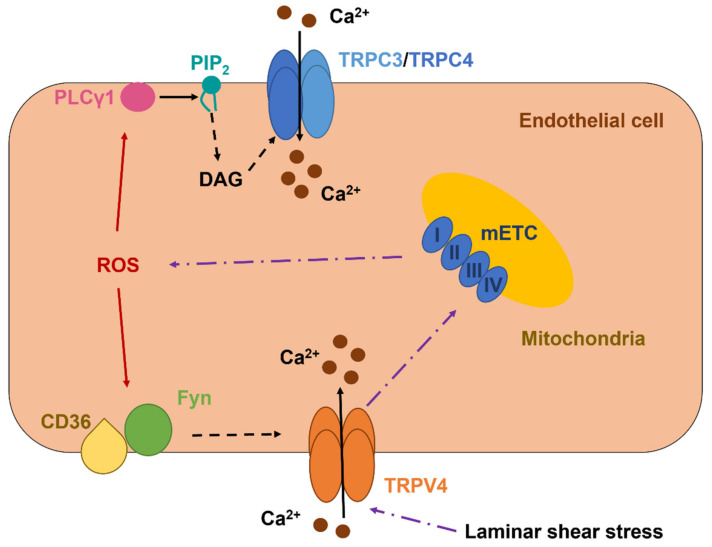
ROS activates TRPC3/TRPC4 heterotetramers and TRPV4 in vascular endothelial cells. ROS may activate endothelial TRPC3/TRPC4 heterotetramers and TRPV4 by exploiting two distinct mechanisms. ROS could stimulate PLCγ1 to cleave DAG from the minor membrane phospholipide, PIP_2_, thereby gating the TRPC3/TRPC4 heterotetramer. ROS could be detected by Fyn, which is required to activate TRPV4 in a redox-sensitive manner. The physical association between Fyn and TRPV4 is maintained by CD36. Laminar shear stress may boost the mitochondrial production of ROS by stimulating TRPV4-mediated extracellular Ca^2+^ entry.

TRPC3 is a DAG-sensitive channel that presents a P_Ca_/P_Na_ of 1.62 and mediates extracellular Ca^2+^ entry upon PLC recruitment by G_q/11_PCRs and TKRs [7,206]. TRPC3-dependent increase in endothelial [Ca^2+^]_i_ controls proliferation, migration, tube formation, barrier permeability, and generation of chemical (e.g., NO) and electrical (i.e., EDH) vasorelaxing signals [15,106,204]. Early work showed that t-BOOH activated TRPC3 to mediate a nonselective cation current in PAECs (Figure 2 and Table 2) [207]. A follow-up investigation revealed that TRPC3 may assemble with TRPC4 to form a heterodimer that is activated by intracellular ROS [208]. The functional role of this redox-sensitive TRPC3/TRPC4 heteromeric channel has not been assessed, but it could be implicated in angiogenesis [209]. ROS signaling is unlikely to exert a direct modulation on either TRPC3 or TRPC4 [60]. However, Groschner’s group (the same group) demonstrated that t-BOOH-mediated activation of the TRPC3/TRPC4-mediated current was sensitive to PLC inhibition [210]. This observation suggests that intracellular ROS could stimulate PLCγ1 to release DAG from PIP_2_, thereby inducing DAG-dependent activation of TRPC3 (Figure 2) [210].

### 5.2. The Role of TRPV1 as a Novel Sensor in Redox Signaling in Vascular Endothelial Cells

**Figure 3:**
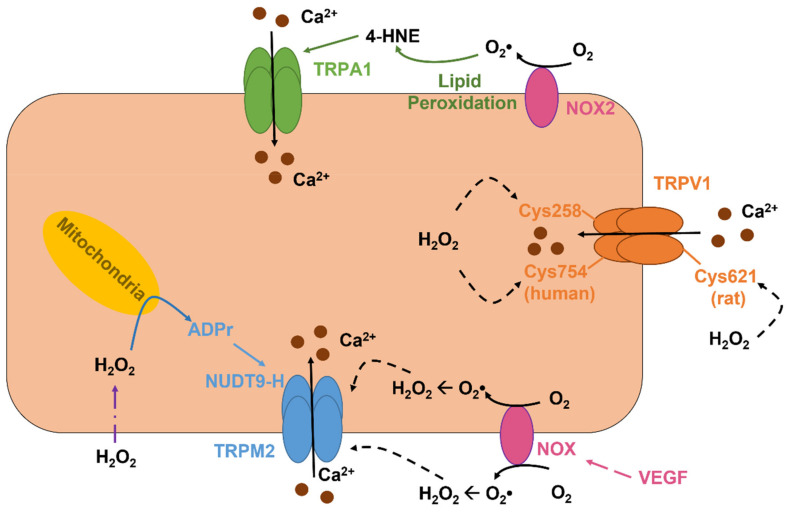
ROS activate endothelial TRPA1, TRPV1, and TRPM2. H_2_O_2_ may directly activate TRPV1, although the underlying mechanism may vary depending on the species and involves the cytosolic Cys258 and Cys274 and the extracellular Cys621 in the human and rat proteins, respectively (please see the text for further explanation). H_2_O_2_ may indirectly activate TRPM2 by inducing the mitochondrial production of ADPr, which binds to the COOH terminal NUDT9-H motif and gates the channel. VEGF-induced NOX2 activation may lead to TRPM2 activation upon intracellular ROS production. NOX2-derived O_2_•^−^ may induce lipid membrane peroxidation and thereby promote 4-HNE formation through the Fenton reaction. 4-HNE, in turn, stimulates TRPA1 to mediate extracellular Ca^2+^ entry.

TRPV1 is a polymodal channel that can integrate both physical and chemical stimuli and shows a P_Ca_/P_Na_ of 9.6 that renders this channel able to regulate multiple endothelial functions, ranging from angiogenesis to vasodilation, as recently reviewed in [3]. TRPV1 may be gated by a variety of physical and chemical stimuli, such as noxious heat (>42 °C), a decrease in extracellular pH, spider-derived vanillotoxins, agonists of plant origin (e.g., capsaicin), and fatty acids conjugated with amines (e.g., anandamide) [3,5]. Although not explicitly recognized as a sensor of endothelial redox signaling [60], TRPV1 may also be activated by oxidant stress (Figure 3) [211,212,213], although the underlying mechanism varies among species. H_2_O_2_ activates the rat TRPV1 by oxidizing the extracellular Cys621 (Figure 3), which may serve as a switch to open the channel pore [211], whereas chicken TRPV1 is activated in a graded manner by the oxidation of multiple Cys residues that are located at the NH_2_ and COOH termini [212]. Furthermore, H_2_O_2_-induced activation of the chicken TRPV1 impinges on COOH-terminal dimerization through intersubunit disulfide bond pairing [214]. The sensitivity of human TRPV1 to redox signaling is finely tuned by Cys258 and Cys754 (Figure 3), which are, respectively, positioned at the NH_2_ and COOH termini of the channel protein and mediate the formation of an intersubunit disulfide bond that is required to maintain the heterotetramer stability [215]. However, one of the Cys258 of the TRPV1 dimer is engaged by the disulfide pairing, while the other Cys-258 retains a free reactive thiol that can be oxidized by H_2_O_2_ and thereby induce the conformational change leading to TRPV1 activation [215]. A recent investigation demonstrated that TRPV1 may sense redox signaling in mouse coronary artery endothelial cells (MCAECs) and BAECs (Table 2) [216]. DelloStritto et al. revealed that acute exposure to H_2_O_2_ elicits nonselective cation currents in these cells and induce vasodilation of mouse coronary artery, thereby leading to an increase in local blood perfusion. In addition, H_2_O_2_ potentiated the bioelectrical signals induced by capsaicin, a specific TRPV1 agonist [216]. Intriguingly, prolonged (1 h) pretreatment with H_2_O_2_ blunts both capsaicin-induced nonselective cation currents in BAECs and coronary vasodilation in mouse [216]. This observation suggests that endothelial TRPV1 signaling could be severely impaired by cardiovascular risk factors associated with enhanced oxidant stress [3].

### 5.3. The Role of TRPV4 in Vascular Endothelial Cells: A Sensor and an Inducer of Redox Signaling

TRPV4 is a another polymodal channel that presents a P_Ca_/P_Na_ ranging between 6 and 10 and, therefore, controls crucial Ca^2+^-dependent vascular functions, e.g., angiogenesis, permeability, NO release, and EDH [60,96,217,218]. In addition, TRPV4 is expressed and mediates proangiogenic Ca^2+^ signals in circulating ECFCs [97,122]. TRPV4 is gated by a multitude of cues, including a moderate increase in temperature (>27 °C), pulsatile stretch, laminar shear stress, hypotonic cell swelling, arachidonic acid, EETs, and anandamide [217,218]. Furthermore, the endothelial TRPV4 is finely tuned by G_q/11_PCRs/PLC signaling, as extensively reviewed in [7,25,217]. TRPV4 was found to support H_2_O_2_-induced increase in [Ca^2+^]_i_ in both mouse and human mouse pulmonary microvascular endothelial cells (Table 2) [219]. The Ca^2+^ response to H_2_O_2_ required the basal phosphorylation of TRPV4 by the Src kinase Fyn, which may serve as the redox sensor responsible for TRPV4 activation (Figure 2) [220], and was able to increase barrier permeability [219]. A follow-up report revealed that the fatty acid transporter, CD36, is indispensable to associate Fyn to the plasma membrane and maintain H_2_O_2_-induced extracellular Ca^2+^ entry through TRPV4 in lung microvascular endothelial cells (Figure 2) [221]. Intriguingly, TRPV4 activation by laminar shear stress may also induce the mitochondrial production of H_2_O_2_ and O_2_•^−^ in HAECs (Figure 2) [222,223]. The subsequent release of H_2_O_2_, in turn, is responsible for flow-induced vasodilation in human coronary resistance arteries [222,224].

### 5.4. The Role TRPM2 as an Indirect Sensor of Redox Signaling in Vascular Endothelial Cells

TRPM2 is the first TRP isoform that has been shown to serve as ROS sensor [225,226] and is widely expressed in vascular endothelial cells [60]. TRPM2 is a nonselective cation channel that displays a linear current-to-voltage relationship with a reversal potential (E_rev_) of ~0 mV and a P_Ca_/P_Na_ of ~0.3-0.9 [227]. TRPM2-mediated extracellular Ca^2+^ entry regulates a variety of endothelial functions, ranging from the control of vascular permeability and blood pressure to angiogenesis [5,7,228]. TRPM2 can be indirectly activated by extracellular H_2_O_2_ that accumulates during tissue inflammation and damage. H_2_O_2_ is freely permeable across the plasma membrane, although it can also pass through specific aquaporins (e.g., aquaporins 3, 5, 8, 9, and 11) [194,229], and, once in the cytosol, can induce the mitochondrial production of the second messenger ADP ribose (ADPr), through a mechanism that is likely to involve NAD metabolism by PARP1 (Figure 3) [230,231,232]. ADPr, in turn, binds to the nudix box phosphohydrolase enzymatic domain (NUDT9-H) that is located in the COOH terminal of the channel protein and thereby leads to TRPM2 activation (Figure 3) [226,230]. A local increase in submembrane Ca^2+^ concentration is required to sustain ADPr-induced TRPM2 activity over time [233]. In contrast, the long-lasting view that TRPM2 could also be activated by cADPr binding to the NUDT9-H domain has been refuted by recent evidence [234,235]. TRPM2 mediates H_2_O_2_-induced extracellular Ca^2+^ entry in endothelial cells from multiple vascular districts (Table 2) [228]. Malik’s group was the first one to report the role of TRPM2 in H_2_O_2_-evoked nonselective cation current and Ca^2+^ influx in HPAECs, thereby causing a decrease in endothelial permeability (Table 2) [236]. This observation led to the concept that aberrant TRPM2 activation could be involved in edema formation and blood-brain barrier (BBB) disruption during prolonged oxidative stress [60]. Subsequent work showed that, in mouse pulmonary artery endothelial cells, VEGF activated NOX2 to elicit the ROS-dependent activation of TRPM2 (Figure 3) [237]. The TRPM2-dependent increase in endothelial [Ca^2+^]_i_, in turn, stimulated c-Src to phosphorylate VE-cadherin, thereby promoting its internalization and disassembly of adherens junctions, which is a crucial step in endothelial cell migration [237]. In agreement with this observation, a subsequent report showed that TRPM2 was activated by NOX4-dependent generation of intracellular ROS to sustain platelet lysate-induced Ca^2+^ signals and cell migration in bEND5 cells [197].

### 5.5. The Role of TRPM4 in ROS-Induced Angiogenesis

TRPM4 is a Ca^2+^-activated, Ca^2+^-impermeable nonselective cation channel that presents a P_Ca_/P_Na_ of 0.09 [7] and control endothelial cell permeability and sprouting angiogenesis [5]. At the negative resting membrane potential (V_M_) of vascular endothelial cells [106], extracellular Na^+^ entry through TRPM4 depolarizes V_M_ to dampen the driving force sustaining Ca^2+^ influx into the cytosol and thereby prevents the cytotoxic Ca^2+^ overload [5]. Thus, TRPM4 activation could be crucial for the onset and maintenance of the most appropriate Ca^2+^ waveform sustaining endothelial signaling in response to specific chemical and physical cues [5]. A recent investigation showed that TRPM4 was required by H_2_O_2_ (1-10 µM) to induce HUVEC depolarization and sustain fetal bovine serum (FBS)-induced migration, proliferation, and adhesion (Table 2) [238]. TRPM4 protein is not known to possess ROS-sensitive reactive thiols [60]. Therefore, it is likely that H_2_O_2_ recruits TRPM4 by inducing an increase in endothelial [Ca^2+^]_i_. In this regard, FBS has long been known to stimulate proliferation and proliferation in a Ca^2+^-dependent manner [239,240]. Future work will have to assess whether TRPM4 activation prevents FBS-induced cytosolic Ca^2+^ overload in HUVECs.

### 5.6. The Role of ROS-Sensitive Endothelial TRPA1 in Dilation of Cerebral Arteries and in Neurovascular Coupling

TRPA1 provides another example of a highly versatile endothelial channel that is more permeable to Ca^2+^ than Na^+^ (P_Ca_/P_Na_ = 7.9) and can be activated by an array of stimuli, including the pungent dietary agonists allicin (garlic), cinnamaldehyde (cinnamon), and allyl isothiocyanate (mustard) [7,60]. TRPA1 is widely expressed in vascular endothelial cells lining cerebral pial arteries and parenchymal arterioles, but it is not detectable in the arterial endothelium of other vascular districts [241]. Intriguingly, TRPA1 is highly enriched in the endothelial membrane projecting through the internal elastic lamina to connect with the overlying VSMCs through heterocellular myoendothelial gap junctions (MEGJs) [241]. Herein, TRPA1 colocalizes in nanometer proximity with NOX2 and the intermediate- and small-conductance Ca^2+^-activated K^+^ (IK_Ca_/SK_Ca_) channels that mediate EDH [77]. Earley’s group demonstrated that NOX2-derived O_2_•^−^ induced lipid membrane peroxidation followed by 4-HNE formation through the Fenton reaction. 4-HNE, in turn, stimulated TRPA1 to mediate submembrane Ca^2+^ sparklets that evoked dilation of cerebral arteries by recruiting IK_Ca_/SK_Ca_ (Table 2) [77]. A follow-up study further revealed that TRPA1 is also expressed in brain capillary endothelial cells and may sustain the hemodynamic response to prolonged sensory stimulation [242]. Neurovascular coupling (NVC), also known as functional hyperemia, is the mechanism whereby an increase in neuronal activity (NA) leads to a local increase in cerebral blood flow (CBF) to match the increasing neuronal demand for O_2_ and glucose [24,25]. An increase in [Ca^2+^]_i_ is required by cerebrovascular endothelial cells to regulate a myriad of functions, including BBB permeability [243] and release of vasoactive mediators [24]. Thakore et al. found that TRPA1 can be activated during prolonged neuronal activity by metabolically active neurons [244] or astrocytes [245,246]. TRPA1-mediated extracellular Ca^2+^ entry causes an increase in [Ca^2+^]_i_ that triggers a vasorelaxing signal slowly propagating back from the capillary bed to the upstream precapillary arterioles due to the Ca^2+^-dependent release of ATP via pannexin 1 (Panx1). ATP, in turn, gates P2X receptors to elevate the [Ca^2+^]_i_ in the adjoining cells, thus initiating a spreading intercellular Ca^2+^ wave that impinges on Ca^2+^-dependent Panx1 activation and paracrine ATP signaling [242]. Once this propagating Ca^2+^ sweep reaches the postarteriole transitional segment, the local increase in endothelial [Ca^2+^]_i_ is transformed into a hyperpolarizing electrical signal, i.e., EDH, by the Ca^2+^-dependent recruitment of IK_Ca_/SK_Ca_ channels, thereby vasodilating the upstream intraparenchymal arterioles and causing a local increase in CBF [241]. The redox sensitivity of endothelial TRPA1 channels may exert a neuroprotective role during brain stroke [241]. Indeed, hypoxia (pO_2_ of ~10-15 mmHg) was found to promote mitochondrial ROS generation, which was followed by 4-HNE formation and TRPA1-dependent vasodilation of cerebral pial arteries and intraparenchymal arterioles [247]. Therefore, ROS-dependent TRPA1 activation was indispensable to limit ischemic damage to the brain [241,247].

**Table 2:** Representative studies showing the direct effect of ROS on endothelial TRP channels.

ROS	Mechanism of ROS Stimulation	Dose of ROS or of ROS-Generating Enzymes	Endothelial Cell Type	TRP Targeted	Function	Ref.
t-BHQ	Acute exposure	400 µM	PAECs	TRPC3	Unknown	[207,210]
ChOx	Acute exposure	0.5 u/mL	PAECs	TRPC3/TRPC4	Unknown	[208]
H_2_O_2_	Acute exposure	250 µM	MCAECs and BAECs	TRPV1	Vasodilation	[216]
H_2_O_2_	Acute exposure	250 µM	Human and mouse lung microvascular endothelial cells	TRPV4	Barrier permeability	[223]
H_2_O_2_	Acute exposure	0–500 µM	HPAECs	TRPM2	Decrease in barrier permeability, apoptosis	[58,236]
H_2_O_2_	Acute exposure	300 µM	Mouse lung microvascular endothelial cells	TRPM2	Decrease in barrier permeability, neutrophil migration	[36]
H_2_O_2_	Acute exposure	0.5–1 mM	Mouse brain endothelial cells	TRPM2	Aβ_1-40_ -induced endothelial dysfunction	[38]
H_2_O_2_	Acute exposure	Not specified	MAECs	TRPM2	Endothelial dysfunction	[54]
H_2_O_2_	Acute exposure	3 mM	H5V	TRPM2	Apoptosis	[248]
H_2_O_2_	Acute exposure	1–10 µM	HUVECs	TRPM4	Migration, spreading, and adhesion	[238]
4-HNE	Acute exposure	5–1000 nM	Mouse brain endothelial cells	TRPA1	Vasorelaxation, neuroprotection, and NVC	[77,242,247]

Abbreviations: BAECs: bovine aortic endothelial cells; CxOx: cholesterol oxidase; HPAECs: human pulmonary artery endothelial cells; PAECs: porcine aortic endothelial cells; HUVECs: human umbilical vein endothelial cells; MAECs: mouse aortic endothelial cells; MCAECs: mouse coronary artery endothelial cells.

## 6. Therapeutic Applications and Pathological Implications of ROS-Induced Endothelial Ca^2+^ Signals

As inferred by the evidence described above, ROS-induced intracellular Ca^2+^ signals regulate a variety of endothelial functions, which may be hampered when ROS overproduction overwhelms the intrinsic antioxidant capacity of vascular endothelial cells. In this conclusive Section, we first discuss the evidence in favor of the therapeutic applications of ROS-dependent endothelial Ca^2+^ signaling to rescue vascular functions. Then, we describe how aberrant and/or chronic oxidant stress may result in an exaggerated increase in endothelial [Ca^2+^]_i_ that may severely compromise vascular signaling.

### 6.1. Exploiting ROS-Induced Endothelial Ca^2+^ Signals to Promote Therapeutic Angiogenesis and Rescue Blood Flow Perfusion

VEGF may impinge on the local and finely tuned intracellular generation of ROS downstream of VEGF receptor-2 (VEGFR-2) to stimulate angiogenesis and restore local blood flow in ischemic tissues [46,52]. Likewise, an increase in [Ca^2+^]_i_ sustains endothelial cell proliferation, migration, and tube formation [26,206]. As outlined above, VEGF-induced proangiogenic Ca^2+^ signals in HAECs are sustained by S-glutathionylation of SERCA2B Cys674 following NOX4-mediated H_2_O_2_ production [164]. Likewise, VEGF-induced extracellular Ca^2+^ entry in human lung vascular endothelial cells requires the ROS-dependent activation of TRPM2, and this signaling pathway contributes to VEGF-dependent postischemic angiogenesis in a mouse model of hindlimb ischemia [237]. These preliminary observations suggest that ROS-induced endothelial Ca^2+^ signaling could represent a promising strategy to achieve therapeutic angiogenesis in ischemic disorders. In accordance with this hypothesis, platelet lysate-derived intracellular Ca^2+^ signals, which are triggered by NOX4, drive bEND5 cell migration in vitro [197] and this is consistent with the notion that this mixture of growth factors and chemokines and cytokines can be locally injected to induce revascularization of ischemic tissues [4]. Similarly, buckwheat-honey-induced, H_2_O_2_-dependent intracellular Ca^2+^ signals exerted a chemotactic effect on bEND5 cells [198]. Of note, local honey delivery through cryogels, hydrogels, and electrospun scaffolds has been presented as a promising strategy to induce wound healing and tissue regeneration [249]. It was shown that transient delivery of low-to-moderate doses of H_2_O_2_ (0.1-100 µM) may promote proliferation, migration, and tube formation in endothelial cells from different vascular beds [250,251,252], while higher doses induce endothelial cell death [252,253]. Therefore, the tunable release of adequate amounts of H_2_O_2_ by dynamic hydrogel matrices into injured tissues could induce proangiogenic Ca^2+^ signals in local endothelial cells [254,255]. An alternative strategy to exploit ROS-induced endothelial Ca^2+^ signaling for regenerative purposes consists in the optical stimulation of photosensitive conjugated polymers, which generate H_2_O_2_ upon exposure to visible light [3,256]. A recent investigation revealed that optical excitation (525 nm) of the regioregular poly(3-hexyl-thiophene) (rr-P3HT) stimulate ECFC proliferation and tube formation through the H_2_O_2_-dependent recruitment of TRPV1 [257]. TRPV1-mediated extracellular Ca^2+^ entry was, in turn, able to engage the transcriptional program driving angiogenesis by inducing the nuclear translocation of the Ca^2+^-sensitive transcription factor, NF-κB [256,257]. Optical excitation of photosensitive conjugated polymers provides the spatiotemporal resolution required to generate a transient increase in local H_2_O_2_ concentration that can sustain angiogenesis in a Ca^2+^-dependent manner [3,256]. Further work is required to design nanomaterials that are excited by near-infrared light, which may penetrate within the deeper layers of a tissue, and to assess whether other ROS-sensitive TRP channels, e.g., TRPM2 and TRPA1, are recruited downstream of H_2_O_2_. This approach may prove extremely helpful to induce therapeutic angiogenesis in ischemic organs. Intriguingly, it has been shown that hypoxia-induced ROS lead to TRPA1 activation in mouse cerebrovascular endothelial cells and the ensuing TRPA1-mediated vasodilation contributes to halt ischemic damage after stroke (Table 2) [247]. Therefore, recruitment of appropriate TRP channels via local release/production of adequate amounts of ROS could exert more beneficial effects than expected in injured tissues.

### 6.2. Exploiting ROS-Induced Endothelial Ca^2+^ Signals to Treat Cancer

It has long been known that an aberrant increase in [Ca^2+^]_i_ may result in a cytotoxic effect by stimulating several Ca^2+^-dependent modes of cell death, including necrosis and apoptosis [258]. A number of chemotherapeutics were found to induce cell death by inducing an uncontrolled elevation in [Ca^2+^]_i_ [258,259,260]. In addition to promoting tissue regeneration, H_2_O_2_-releasing nanomaterials can exert an anticancer effect by increasing the already high extent of oxidant stress imposed to cancer cells by tumor microenvironment [261,262]. Interestingly, many ROS-sensitive TRP channels are aberrantly expressed in tumor endothelial cells [5,43,263] and could, therefore, transduce the oxidant stress into a cytotoxic increase in [Ca^2+^]_i_. For instance, a recent transcriptional analysis revealed that TRPA1 is upregulated in prostate-cancer-derived endothelial cells (PCECs), but not in those harvested from breast and kidney cancer [263]. Furthermore, PCECs present high levels of TRPV2, which is not directly gated by ROS signaling [60], but mediates H_2_O_2_-induced cytotoxicity in human hepatoma cells [264]. As reviewed in [5,43], the H_2_O_2_-sensitive TRPV4 channel is also upregulated in breast cancer-derived-endothelial cells, while it is downregulated in Lewis lung carcinoma. A number of strategies, including photodynamic therapy [265,266] and H_2_O_2_-releasing and H_2_O_2_-responsive nanomaterials [267,268,269], are seeking to induce prostate and breast cancer cell death through an exaggerated oxidant stress. Future work will have to assess whether ROS-sensitive endothelial TRP channels, such as TRPA1, TRPV1, TRPV2, TRPV4, and TRPM2, contribute to H_2_O_2_-dependent anticancer effect by inducing endothelial cell death and thereby dismantling cancer neovessels. As suggested for cancer cells [270,271], the overexpression of ROS-sensitive TRP channels in tumor, but not healthy, endothelium, could afford a novel opportunity to exploit lower concentrations of ROS to selectively target the tumor microenvironment and to reduce the unwanted off-target effects on tumor-adjacent normal tissues.

### 6.3. Pathological Implications of ROS-Induced Endothelial Ca^2+^ Signaling

Excessive ROS generation may result in endothelial dysfunction and compromise the physiological control of vascular function and architecture in multiple cardiovascular diseases, such as ischemia/reperfusion, atherosclerosis, hypertension, diabetes, infection, and inflammation [44,46,127,272]. This evidence led to the proposal that an exaggerated increase in [Ca^2+^]_i_ sustains ROS-induced endothelial injury [60,68,127]. For instance, macrophage-derived ROS were shown to induce endothelial apoptosis by mobilizing the InsP_3_-sensitive ER Ca^2+^ pool, thereby promoting mitochondrial depolarization and recruiting both the intrinsic and extrinsic caspase pathways [273]. Likewise, ROS produced upon ischemia-reperfusion injury in the heart cause endothelial cell death by promoting InsP_3_-dependent mitochondrial Ca^2+^ overload, mPTP opening and release of cytochrome c in the cytosol [274].

In the present Section, we describe the most recent findings that hint at intracellular Ca^2+^ signaling as one of the main executors of ROS-dependent endothelial dysfunction.

#### 6.3.1. The Role of ROS-Induced Endothelial Ca^2+^ Signaling in the Inflammatory Response

Systemic accumulation of bacterial endotoxins such as lipopolysaccharide (LPS) signals the disruption of the endothelial barrier through an increase in [Ca^2+^]_i_ that causes endothelial cell contraction [37,68]. A number of studies demonstrated that LPS elicits intracellular Ca^2+^ signals in vascular endothelial cells [22,37], although not in circulating ECFCs [275]. Gandhirajan et al. revealed that Toll-like receptor 4 (TLR4) activation by LPS results in repetitive Ca^2+^ transients in mouse pulmonary artery endothelial cells [22]. LPS-induced intracellular Ca^2+^ oscillations were driven by NOX2-dependent H_2_O_2_ production, which induced the dynamic interplay between InsP_3_R2-dependent ER Ca^2+^ release and STIM1-dependent SOCE [22]. The oscillatory Ca^2+^ signal led to the nuclear translocation of NFAT, which, in turn, was required to drive the expression of proinflammatory genes responsible for LPS-induced increase in vascular permeability [22]. Moreover, LPS-induced intracellular Ca^2+^ oscillations could result in endothelial cell necroptosis through the Ca^2+^-dependent upregulation of receptor-interacting protein 3-dependent (RIP3) [22]. The pharmacological blockade of SOCE with the pyrazole derivative, BTP-2 [199], hindered LPS-dependent vascular leakage and pulmonary edema [22], thereby suggesting that ROS-dependent Ca^2+^ signaling represents a promising target to halt endothelial dysfunction. An alternative signaling pathway whereby ROS signaling may induce pulmonary vascular permeability and inflammation is through TRPC6 activation [59]. Endothelial NOX2 is activated at the beginning of lung ischemia-reperfusion injury, thereby causing robust increase in intracellular H_2_O_2_ levels. H_2_O_2_, in turn, recruits PLCγ to stimulate DAG production and subsequent TRPC6-mediated cytosolic Ca^2+^ overload. Moreover, H_2_O_2_ inhibits DAG kinase η, thereby preventing DAG metabolism and further increase sub-membranal DAG concentration [59]. This mechanism strongly resembles the gating of TRPC3/TRPC4 heterodimers by physiological ROS signaling (Section 5.1 and Table 1).

#### 6.3.2. The Role of ROS-Induced Endothelial Ca^2+^ Signals in Metabolic Disorders

Endothelial cells chronically exposed to excessive amounts of glucose and free fatty acids in the blood, as observed in diabetes and obesity, undergo severe oxidant stress that ultimately results in endothelial dysfunction and leads to severe cardiovascular diseases [276,277,278]. ROS-induced intracellular Ca^2+^ signals could play a crucial role in endothelial dysfunction in metabolic disorders [8,279]. As anticipated in Section 4.3, prolonged hyperglycemia (30 mM for 96 h) upregulates SOCE in HUVECs in a ROS-dependent manner [202]. The subsequent Ca^2+^ entry via Orai1 may elicit endothelial cell apoptosis and mitochondrial depolarization by engaging the tyrosine kinase pp60^src^ [202]. In agreement with this observation, the increased expression of STIM1-2 and Orai1-3 has been reported in aortic endothelial cells harvested from human diabetic patients and from streptozotocin-induced and Akita (C57BL/6-Ins2^Akita^/J) diabetic mice [173]. Intriguingly, hyperglycemia-impaired agonist-induced NO release from endothelial cells in cultured human vascular endothelial cells [280], in mouse models of diabetes [281], and in human patients [279], although Orai1-mediated SOCE is the main responsible for the recruitment of the Ca^2+^/CaM-dependent eNOS [8]. To explain this apparent controversy, it has been proposed that enhanced SOCE results in the engagement of the Ca^2+^-sensitive calpain [282,283], which reduces NO bioavailability by dissociating the regulatory protein heat shock protein 90 from eNOS [281,284]. In addition, the endothelial caveolar subcellular domain may be altered in type 2 diabetes and obesity [8]. Caveolae represent Ω-shaped invaginations of the plasma membrane that place Orai1 channels in physical contiguity with their downstream Ca^2+^-dependent decoders, such as eNOS [24]. The derangement of the caveolar signaling platform could uncouple eNOS from its main physiological Ca^2+^ source in endothelial cells lining the lumen of large vessels [8,106,166], where NO-dependent vasodilation predominates over other vasorelaxing mechanisms [84], in metabolic disorders [8]. Furthermore, the enhanced SOCE could boost NOX activity [285,286], thereby increasing the intracellular levels of O_2_•^−^, which scavenges NO and further impairs NO-dependent vasodilation [280,287]. An additional mechanism whereby oxidant stress imposed on vascular endothelium by hyperglycemia could increase extracellular Ca^2+^ entry in response to physiological agonists is via SERCA2B inhibition [33,162,163]. Berra-Romani and coworkers reported that SERCA2B protein is upregulated in the native endothelium of excised rat aorta harvested from obese Zucker diabetic rats [33]. Nevertheless, SERCA2B activity was downregulated by intracellular ROS, thereby failing to sequester extracellular Ca^2+^ incoming through store-operated channels and exaggerating the Ca^2+^ response to NO-producing agonists [33]. Paradoxically, a recent investigation demonstrated that ROS-dependent endothelial cell apoptosis in small resistance arteries is lower in male mice fed with a Western-style diet (WS) enriched in carbohydrates and fat [288], which would per se contribute to insulin resistance, obesity, and heart failure. Endothelial resilience to WD-induced oxidative stress is associated to the downregulation of TRPV4-mediated extracellular Ca^2+^ entry [61,288]. Interestingly, a reduction in endothelial TRPV4 channel expression and/or activity could be also implicated in microvascular adaptation to aging-induced oxidative stress on the tunica intima [289]. As anticipated in Section 5.2, prolonged exposure to oxidative stress could impair TRPV1 activity in vascular endothelial cells and thereby affect vasoreactivity [216]. A follow-up report by DelloStritto et al. showed that 4-HNE, a byproduct of lipid peroxidation, reduces capsaicin-induced Ca^2+^-permeable currents and intracellular Ca^2+^ signals in MCAECs and capsaicin-evoked vasodilation in mouse coronary arteries [78]. This effect required 4-HNE-induced oxidation of Cys-621, which is located in the pore helices, and is likely to underlie the inhibitory effect of prolonged exposure to oxidative stress on the signaling pathways regulated by TRPV1 in vascular endothelium [78]. Therefore, it has been hypothesized that TRPV1-dependent increase in coronary blood flow in a mouse model of diabetes is blunted by 4-HNE-mediated post-translational modifications [78,216].

#### 6.3.3. The Role of TRPM Channels in ROS-Induced Endothelial Dysfunction

ROS, which may be generated in excessive amounts by macrophages and polymorphonuclear neutrophils (PMNs) at sites of inflammation and injury, can induce either endothelial cell death or endothelial hyperactivation with consequent disruption of the vascular barrier [61,68,127]. As anticipated in Section 5.4, the pioneering study by Hecquet et al. provided the first evidence that extracellular Ca^2+^ entry in HPAECs through TRPM2 mediated H_2_O_2_-dependent endothelial hyperpermeability (Table 2) [236]. A follow-up study showed that TRPM2-induced intracellular Ca^2+^ overload in human and mouse pulmonary endothelial cells was also able to induce apoptosis by activating caspase-3 (Table 2) [58]. In agreement with these observations, TRPM2 may drive the Ca^2+^-dependent dismantling of the lung endothelial barrier by particulate matter (PM) [290,291]. PM-induced increase in intracellular H_2_O_2_ levels led to TRPM2 activation, followed by the Ca^2+^-dependent recruitment of calpain, degradation of tight junctions Zonula occludens-1 proteins, and endothelial barrier disruption [290]. More recently, TRPM2 was found to mediate the intracellular Ca^2+^ overload evoked by high doses of H_2_O_2_ (3 mM) also in the murine cardiac microvascular endothelial cell line, H5V (Table 2) [248]. TRPM2-mediated extracellular Ca^2+^ entry caused the activation of caspase-8, caspase-9, and caspase-3, thereby causing H_2_O_2_-induced endothelial cell apoptosis (Table 2) [248]. Likewise, TRPM2 was involved in H5V cell death induced by the inflammatory cytokine, tumor necrosis factor-α (TNF-α), which has long been known to induce ROS formation in vascular endothelial cells [292]. TRPM2 was also found to mediate H_2_O_2_-induced cell death in brain microvascular endothelium [228]. In addition to providing the building blocks for the BBB [293], brain microvascular endothelial cells are emerging as crucial regulators of neuronal activity and cerebral blood flow under both physiological and pathological conditions [24,25,41]. Iadecola’s group first showed that amyloid β_1-40_ (Aβ_1-40_), whose extracellular accumulation on brain microvessels is now regarded as the primary trigger of the pathogenic pathways leading to neuronal damage and dementia [294], may induce endothelial dysfunction by promoting TRPM2-mediated cytosolic Ca^2+^ overload [38]. In accord, Aβ_1–40_ activated CD36 on the plasma membrane, thereby stimulating NOX2-dependent O_2_•^−^ formation in mouse brain microvascular endothelial cells (Table 2) [38]. O_2_•^−^ may then react with NO, which is constitutively synthesized by brain endothelium [24], to form ONOO^-^ [38]. ONOO^-^-dependent DNA damage results in PARP activation within the nucleus and the subsequent production of ADPr by PARG-mediated cleavage of PAR triggers extracellular Ca^2+^ through TRPM2 [38]. This sustained increase in [Ca^2+^]_i_ is likely to be responsible for endothelial dysfunction and to interfere with the subtle regulation of the Ca^2+^-dependent vasoactive pathways that drive neurovascular coupling [8,38]. For instance, Aβ_1-40_-induced oxidative stress in endothelial cells may inhibit Ach-induced, TRPV4-dependent EDH and vasodilation in cerebral arteries [295]. Furthermore, TRPM2-mediated extracellular Ca^2+^ entry could accelerate mitochondrial oxygen consumption and boost mitochondrial production of O_2_•^−^, which further exacerbates Aβ_1-40_-induced endothelial dysfunction [56]. Furthermore, TRPM2 contributes to methamphetamine (METH)- and HIV-TAT-induced BBB injury [296]. METH and HIV-TAT synergistically caused a remarkable increase in intracellular ROS levels in human brain microvascular endothelial cells. The oxidant stress, in turn, activated TRPM2 to mediate extracellular Ca^2+^ entry, which promoted endothelial cell apoptosis and downregulated the expression of multiple tight junctions proteins, such as occluding and junctional adhesion molecule A (JAMA) and occludin, and of ZO1 [296]. The notion that the endothelial TRPM2 could provide a promising molecular target to halt brain injury by oxidant stress is further suggested by the evidence that a novel peptide inhibitor, tat-M2NX, which prevents ADPr binding to the COOH-terminal NUDT9-H sequence, afforded neuroprotection and reduced brain injury in murine models of brain stroke [39]. A recent investigation revealed that TRPM2 can be recruited by extracellular Ca^2+^ entry through N-methyl-d-aspartate (NMDA) receptors and elicit proinflammatory signaling in brain microglia [297]. Of note, NMDA receptors are also expressed and elicit Ca^2+^-dependent NO production also in cerebrovascular endothelium [298]. Future work might assess whether excessive glutamate release during chronic inflammation also results in aberrant activation of endothelial TRPM2 in brain microcirculation. Furthermore, TRPM2-mediated intracellular Ca^2+^ overload drives apoptosis in mouse PAECs (mPAECs) infected with the H9N2 influenza virus [299]. H9N2 virus-induced DNA damage led to intracellular production of ROS, which activated TRPM2 to promote the Ca^2+^-dependent recruitment of caspase-3/7, mitochondrial depolarization, and endothelial cell apoptosis [299].

TRPM2 may also sustain endothelial damage during acute lung injury (ALI) [272] and metabolic syndrome [228]. For instance, genetic deletion of the endothelial TRPM2 reduced LPS-induced pulmonary endothelial cell death, PMN infiltration in the lungs, and pulmonary inflammatory injury [36,58]. Furthermore, mice conditionally (with tamoxifen) knocked out for endothelial TRPM2 displayed a survival rate of 80% upon intraperitoneal injection of a lethal dose of LPS, while wild-type mice did not survive. PMN interaction with lung vascular endothelial cells caused an increase in intracellular ROS levels, thereby inducing PARP1-dependent ADPr production and TRPM2 activation. TRPM2-mediated extracellular Ca^2+^ entry triggered endothelial barrier dysfunction and favored PMN transendothelial migration through the disassembly of VE-cadherin (Table 2) [36]. Moreover, TRPM2 is emerging as a crucial molecular player in the onset of obesity-associated endothelial insulin resistance, which is likely to arise in response to an elevation in endothelial ROS levels [228]. TRPM2 expression, H_2_O_2_-induced nonselective cation currents, and H_2_O_2_-induced extracellular Ca^2+^ entry significantly increased in MAECs isolated from adult male C57BL/6 mice fed with a high-fat diet (HFD) as compared to those fed with low-fat chow diet (LFD) (Table 2) [54]. Palmitate is a major saturated free fatty acid that induces endothelial dysfunction by promoting NOX-dependent ROS generation and compromising NO release [228]. Sun and colleagues revealed that TRPM2 mediates palmitate-induced H_2_O_2_-dependent extracellular Ca^2+^ influx in MAECs, thus recruiting the CaMKII/PERK/ATF4/pseudokinase tribble 3 (TRB3) cascade, which inhibits insulin-induced eNOS activation, NO production, and aortic vasorelaxation (Table 2) [54]. In addition, TRPM2 has been recently associated to diabetes-induced endothelial dysfunction [300]. Exposure to HG and exogenous delivery of high doses (3 mM) of H_2_O_2_ induced a large elevation in [Ca^2+^]_i_ in HUVECs that was sustained by TRPM2 [300]. This ROS-sensitive influx of Ca^2+^ mobilized lysosomal Zn^2+^ into the mitochondrial matrix, where Zn^2+^ engaged the small GTPase, dynamin-related protein-1 (Drp-1), to promote mitochondrial fission and, therefore, compromise mitochondrial functioning [300], which is a hallmark of diabetes [8,228]. A comprehensive and exhaustive description of the pathological implications of ROS-induced TRPM2 hyperactivation in vascular endothelial cells can be found in [228,301].

Besides TRPM2, TRPM4 may contribute to ROS-induced endothelial injury during inflammation or as side effect of anticancer treatments. For instance, TRPM4-mediated depolarization sustains LPS-induced cell death in HUVECs [302]. Likewise, TRPM4 sustains endothelial injury caused by arsenic trioxide (ATO) [303], a first-line chemotherapeutic drug that can induce severe cardiotoxicity and has, therefore, been discontinued [304]. A recent investigation showed that ATO-induced oxidative stress enhanced TRPM4 expression in HUVECs, which exacerbated TRPM4-mediated depolarization and Na^+^ entry, resulted in cytosolic Ca^2+^ overload, and promoted endothelial cell death [303]. It has long been known that excessive Na^+^ entry through TRP channel drives reversal of NCX, thereby triggering a massive elevation in [Ca^2+^]_i_ in vascular endothelial cells [16,174,305]. Therefore, future work will have to assess whether the reverse (Ca^2+^ entry) mode of NCX contributes to ATO-induced TRPM4-dependent cytotoxic Ca^2+^ signaling in HUVECs.

#### 6.3.4. The Role of TRPV4 in Pulmonary Arterial Hypertension

PAH is a life-threatening disorder consisting in a progressive increase in pulmonary vascular resistance, which can ultimately lead to right heart failure and patient’s death. PAH is triggered by endothelial injury, which paves the way to the emergence of apoptosis-resistant and hyperproliferative endothelial cells that display impaired release of vasorelaxing mediators and contribute to the formation of occlusive intimal lesions [306,307]. In addition, pulmonary-resident ECFCs could support the proliferative angiopathic process in PAH [308]. Aberrant ROS-dependent endothelial TRPV4 activity has been coupled to PAH [309]. An insightful investigation conducted on a mouse model of PAH revealed that, although TRPV4 protein is not upregulated in lung microvascular endothelial cells, mitochondrial-derived ROS enhance TRPV4-mediated extracellular Ca^2+^ entry, thereby boosting endothelial cell proliferation and migration [31]. A follow-up study further showed that extracellular Ca^2+^ influx through TRPV4 exacerbated mitochondrial fission and fragmentation and decreased mitochondrial respiration [310]. While it is unclear whether CD36 is also implicated in TRPV4 activation by mitochondrial ROS, the pharmacological blockade of TRPV4 could represent a promising strategy to treat PAH [309].

## 7. Conclusions

While the mechanisms shaping the increase in [Ca^2+^]_i_ and ROS production in vascular endothelial cells have been widely investigated, the complex interplay between such two highly versatile signaling pathways is far from being fully dissected. A large body of investigations was devoted to ascertaining the effect of ROS on endothelial TRP channels, while it is still unclear whether ROS engage SOCE in vascular endothelium. Since SOCE plays a pivotal role in the regulation of endothelial Ca^2+^ homeostasis by reloading the ER with Ca^2+^ and maintaining long-lasting Ca^2+^ signals, assessing this issue is of compelling relevance. Similarly, a thorough investigation is necessary to understand the molecular mechanisms whereby ROS (and, of course, which ROS species) control endothelial InsP_3_Rs and whether this mode of regulation changes across the vascular beds or in the presence of pathological conditions enhancing the oxidative stress imposed on the endothelial monolayer. Future work is also necessary to assess whether and which NOX isoform contributes (along with PLC) to trigger the Ca^2+^ response to extracellular stimuli by providing the surge of ROS that sensitize InsP_3_Rs to the accompanying increase in cytosolic InsP_3_ levels and/or to ambient Ca^2+^. Finally, the pathophysiological role of ROS-induced Ca^2+^ signals in circulating ECFCs is still largely unclear and deserves to be more deeply unraveled due to the reduction in ECFCs’ proangiogenic activity in cardiovascular disorders associated to oxidative stress. This wealth of information could pave the way to design alternative treatments to interfere with the life-threatening interconnection between endothelial ROS and Ca^2+^ signaling under multiple pathological conditions.

## Data Availability

Not applicable.
